# Phenotypic Clustering of Addictions Reveals Impulsivity Links to Internalizing Disorders: A Network Analysis

**DOI:** 10.31083/AP49511

**Published:** 2026-03-09

**Authors:** Ardıl Bayram Şahin, Yusuf Huseyin Berrak, Duru Besen, Hilal Askin, Hale Yapici Eser

**Affiliations:** ^1^Graduate School of Health Sciences, Koç University, 34010 Istanbul, Türkiye; ^2^Research Center for Translational Medicine (KUTTAM), Koç University, 34010 Istanbul, Türkiye; ^3^School of Medicine, Koç University, 34010 Istanbul, Türkiye; ^4^Department of Psychiatry, School of Medicine, Koç University, 34010 Istanbul, Türkiye

**Keywords:** addictive behavior, substance-related disorders, depression, anxiety, stress, psychological, impulsivity, anhedonia, network analysis

## Abstract

**Background:**

Substance-related and behavioral addictions frequently co-occur and are increasingly conceptualized as part of an interconnected psychopathological framework. Addictive behaviors are linked to internalizing symptoms, including depression, anxiety, and stress, through shared vulnerability. Although impulsivity, anhedonia, and stress-related processes have been proposed as transdiagnostic factors, their relative roles as central or bridging mechanisms remain unclear. This study aimed to clarify the network structure of multiple addictions by jointly modeling substance-related and behavioral addictions with multidimensional psychological features.

**Methods:**

A total of 169 university students completed standardized self-report measures assessing four substance-related addictions (alcohol, tobacco, cannabis, cocaine) and six behavioral addictions (gambling, shopping, gaming, eating, sexual activity, and work), alongside measures of impulsivity, anhedonia, depression, anxiety, stress, chronic stress, and childhood adversities. Three Extended Bayesian Information Criterion Graphical Least Absolute Shrinkage and Selection Operator (EBICglasso) network models were estimated: (i) an addiction-only network, (ii) an addictions-psychometric features network, and (iii) a focused network integrating selected addictions with affective symptoms, stress, and adversities. Centrality, bridge indices, and predictability were evaluated.

**Results:**

Substance-related and behavioral addictions formed a coherent and interconnected network, with alcohol (0.89), sexual activity (0.77), and cannabis (0.70) emerging as the most central hubs. Impulsivity and anxiety functioned as the primary bridge nodes linking addictive behaviors with internalizing symptomatology, with anxiety exhibiting the highest bridge strength (0.29), followed by impulsivity (0.20). The affective distress cluster, comprising depression, anxiety, and stress, demonstrated the strongest internal connectivity and the highest predictability (R^2^), with explained variance values of R^2^ = 0.33 for depression and R^2^ = 0.30 for both anxiety and stress. Stress exposure and childhood adversity showed cross-domain connections involving sexual activity and alcohol use.

**Conclusion:**

This study models substance-related and behavioral addictions together with internalizing symptoms, impulsivity, anhedonia, and stress-related factors to examine how addictive behaviors are organized within an interconnected network. Alcohol, cannabis, and sexual activity showed relatively high connectivity, while impulsivity and anxiety were consistently positioned as bridge variables linking addictive behaviors with affective distress. These findings provide a descriptive transdiagnostic framework in which shared regulatory traits emerge as key organizing features of addiction comorbidity; however, their potential clinical relevance remains hypothesis-generating and requires validation in longitudinal and clinically diagnosed samples.

## Main Points

1. Substance-related and behavioral addictions form a coherent and interconnected network structure rather than functioning as isolated conditions in this non-clinical sample.

2. Alcohol, cannabis, and sexual activity occupy relatively central positions within the addiction network.

3. Impulsivity and anxiety emerge as key transdiagnostic bridge nodes linking addictive behaviors with internalizing symptoms.

4. Depression, anxiety, and stress form a tightly interconnected affective core across network models.

5. Stress exposure and early adversity show selective associations with specific addictions, indicating heterogeneous vulnerability profiles.

## 1. Introduction

Substance use disorders, together with behavioral addictions, are highly prevalent conditions in the general population and represent a growing clinical and public health concern [1]. With the evolution of diagnostic systems, these disorders have begun to be more clearly conceptualized and systematically defined. Diagnostic and Statistical Manual of Mental Disorders, Fifth Edition (DSM-5) reclassified gambling disorder under substance-related and addictive disorders and introduced internet gaming disorder as a condition warranting further research, while International Classification of Diseases, Eleventh Revision (ICD-11) formally established “disorders due to addictive behaviors” as a distinct category, recognizing gaming disorder as a diagnosable condition [2, 3]. On the other hand, substance and behavioral addictions often cluster together, and the presence of multiple addictions is associated with worse clinical outcomes [4]. In the Alberta adult sample, seven distinct addiction profiles were identified, with nearly 21% of individuals exhibiting two or more concurrent addictive behaviors [1]. Males were overrepresented in the tobacco and sexual activity clusters, while females were overrepresented in the shopping clusters; subjective well-being was lowest in the shopping, tobacco, sexual activity, and multiple addiction clusters and relatively higher in the working and overeating clusters [1]. Similarly, a large-scale school screening of Tunisian adolescents reported that both substance and behavioral addictions were prevalent and co-occurred, particularly gaming, and problematic social media use; tobacco-alcohol and tobacco-alcohol-substance combinations clustered more frequently than expected [5].

In addition, evidence increasingly suggests that substance and behavioral addictions are systematically linked to internalizing symptomatology, including depression, anxiety, and chronic stress [6, 7]. The mechanisms explaining the relation of addictions with internalizing psychopathology are still fragmented, limiting our understanding of shared vulnerability pathways and clinical trajectories. Childhood adversity was included as a theoretically grounded, developmentally salient vulnerability factor with well-established links to affective distress, impulsivity, and addiction-related outcomes. The selection of this variable was theory-driven rather than exhaustive, and other potential risk factors were not included due to scope constraints.

The common neurobiological ground of addiction lies in heightened incentive salience within reward circuits combined with weakened prefrontal inhibitory control. This dual imbalance manifests phenotypically as increased impulsivity and reduced capacity to experience pleasure. Importantly, trait and state impulsivity appear to diverge, and measurement approaches vary across self-report and task-based paradigms, reinforcing the need for integrative analytical models [8]. Impulsivity, reflecting dysregulation between reward-related and executive control systems, has been proposed as a transdiagnostic vulnerability factor underlying addictive behaviors [8]. Furthermore, traumatic life experiences and sustained stress exposure may operate as transdiagnostic vulnerability factors, facilitating both the onset and maintenance of addictive behaviors [9]. These variables may act as “bridge symptoms”, linking addiction clusters with internalizing psychopathology. This framework suggests that impulsivity and anhedonia are not merely correlates but may function as structural bridges connecting addictions with stress-, mood-, and trauma-related domains. Supporting evidence by Gomez *et al*. (2025) [10] demonstrated that alcohol clustered with other substances, while gaming and internet use emerged as highly central nodes, with strong interconnections among behavioral addictions. Prevailing approaches to addiction comorbidity have typically emphasized either disorder-specific mechanisms or the role of shared transdiagnostic vulnerabilities, such as impulsivity and affective distress. Building on these perspectives, the present study adopts a network-based framework to examine how multiple substance-related and behavioral addictions are jointly organized and interconnected with key psychological features. Rather than treating shared vulnerabilities as isolated risk factors, this approach conceptualizes comorbidity as an emergent property of an interconnected phenotypic system.

Building on this foundation, the current study aims to examine ten addictions (four substance-related and six behavioral) within a unified network model to elucidate their shared and distinct links with depression, anxiety, perceived and chronic stress, impulsivity, anhedonia, and childhood adversity.

Using a three-stage network analytic approach, this study seeks to assess (i) the topology and clustering among addictions, (ii) the partial associations and bridge nodes between addictions and clinical dimensions, and (iii) the fine-grained links between the highest-impact addictions and clinical subdimensions. These analyses will be complemented by descriptive correlations and gender-based comparisons. By mapping comorbidity patterns and potential transition pathways across addiction types, it is aimed to clarify whether impulsivity and related constructs function as disorder-specific or transdiagnostic bridges. By delineating the structural interactions between addictive behaviors and internalizing psychopathology, this study advances understanding of their shared risk architecture. Identifying high-impact bridge nodes may inform targeted screening strategies and intervention approaches, contributing to more personalized and mechanism-driven clinical care.

## 2. Materials and Methods

### 2.1 Participants and Procedures 

According to the current literature, the minimum sample size required for an effect size of 0.2, a power of 80%, and a significance level of α = 0.05 was calculated as 153 based on the power analysis (G*power version 3.1.9.6; Heinrich Heine University Düsseldorf, Düsseldorf, Germany) [9]. The G*Power analysis was reported solely to contextualize detectable correlation-level effects and was not intended to justify the sample size for network estimation, as traditional power calculations do not generalize to high-dimensional network models. Inclusion criteria were between 18 and 35 years of age, being literate, and willing to participate voluntarily in exchange for lesson credit. Exclusion criteria included any current psychiatric diagnosis or dementia, current symptoms of substance intoxication, unstable medical conditions that could impair consciousness, cognition, or mood, and a history of head trauma. The study sample consisted of Koç University undergraduate students, selected to reduce confounding factors such as chronic medical illnesses and severe psychiatric conditions, ensuring a relatively homogeneous group that enhances internal validity and minimizes the influence of genetic vulnerability and medical comorbidities on the study outcomes. Recruitment was conducted via the Koç University SONA Research Participation Management System between March 27 and April 22, 2025. Before participation, researchers explained the study procedures and reviewed the informed consent, ensuring sufficient time for informed, voluntary participation. Participants completed the questionnaires via an online Qualtrics survey link under the supervision of research assistants. The survey lasted approximately 20–25 minutes and was completed independently in a quiet environment to minimize response bias. Attention-check items (e.g., simple calculation or selection tasks) were embedded to ensure data quality. The assessment included an informed consent form followed by a sociodemographic data form and standardized self-report measures. After completion, participants received individualized feedback via email, and as compensation, students received one course credit for their participation.

### 2.2 Assessment Methods 

Addictive behaviors were assessed using the Brief Screener for Substance and Behavioral Addictions (SSBA), developed by Schluter *et al*. (2018) [11], and subsequently validated by Hodgins *et al*. (2023) [12], which evaluates addiction risk for four substances (alcohol, tobacco, cannabis, and cocaine) and six behavioral domains (gambling, shopping, video gaming, eating, sexual activity, and work). The Turkish version of the SSBA has demonstrated excellent internal consistency in a validity and reliability study, with a Cronbach’s alpha coefficient of 0.94 [13]. For each addiction type, participants responded to four standardized items assessing frequency of problematic engagement over the past 12 months, with response options ranging from “never” to “always”, including “I did not use” and “I do not know/prefer not to say”. Anhedonia was measured using the Snaith-Hamilton Pleasure Scale (SHAPS), originally developed by Snaith *et al*. (1995) [14], a 14-item dichotomous tool assessing recent hedonic capacity, with scores ≥2 indicating clinically significant anhedonia; its Turkish validity was confirmed by Yapici Eser *et al*. (2020) [15].

Impulsivity was assessed using the Barratt Impulsiveness Scale-11 (BIS-11), developed by Patton *et al*. (1995) [16], a 30-item measure rated on a 4-point Likert scale, validated in Turkish by Güleç *et al*. (2008) [17]. Early life adversity was evaluated with the Childhood Trauma Questionnaire-33 (CTQ-33) developed by Bernstein *et al*. (1994) [18], an expanded version of the CTQ-33 incorporating protection and overcontrol dimensions [19]. Chronic stress was assessed using the Chronic Stress Scale (CSS), adapted by Turner R. J. from Wheaton’s scale (1991, 1997) [20, 21, 22]. The 51-item scale is scored from 0 to 2 (total score: 0–102), with higher scores indicating greater stress; its Turkish validity and reliability were established by Yapici Eser *et al*. (2024) [23]. Finally, symptoms of depression, anxiety, and stress were measured using the Depression Anxiety Stress Scale-21 (DASS-21), developed by Lovibond and Lovibond (1995) [24], a 21-item instrument with 7 items per domain, rated on a 4-point scale, with its Turkish validity and reliability established by Sarıçam (2018) [25].

In the current sample, all psychometric measures demonstrated acceptable to good internal consistency. Cronbach’s α coefficients were as follows: SSBA (α = 0.87), DASS-21 (α = 0.91), CSS (α = 0.88), SHAPS (α = 0.76), BIS-11 (α = 0.82), and CTQ (α = 0.87).

### 2.3 Statistical Analysis

Basic analyses were conducted using IBM SPSS Statistics (version 28; IBM Corp., Armonk, NY, USA). Sociodemographic and clinical characteristics of the participants were examined using descriptive statistics. The correlation between the scores of the four substance-related and six behavioral addiction subscales of the SSBA and levels of depression, anxiety, stress, impulsivity, and anhedonia was analyzed. Correlations were examined using Spearman’s rho because SSBA subscale scores are ordinal and did not meet normality assumptions. To control for multiple comparisons, Bonferroni correction, results with *p* < 0.005 were considered statistically significant, whereas results with 0.005 ≤ *p* < 0.05 were considered marginally significant. Cocaine use was excluded from the analyses due to its low prevalence (n = 2) in the sample, which would have yielded unstable parameter estimates in network models.

### 2.4 Network Analysis

Network analysis was conducted following the four-step framework proposed by Epskamp *et al*. (2018) [26]: (i) estimation of the network model, (ii) examination of network structure, (iii) assessment of accuracy and stability, and (iv) evaluation of dimensional structure using community detection and exploratory graph analysis [27, 28, 29]. Analyses were performed in R (version 4.4.1; R Foundation for Statistical Computing, Vienna, Austria) using the packages bootnet (version 1.6; Sacha Epskamp; https://cran.r-project.org/package=bootnet), qgraph (version 1.9.8; Sacha Epskamp *et al*.; https://cran.r-project.org/package=qgraph), EGAnet (version 2.3.0; Hudson Golino; https://cran.r-project.org/package=EGAnet), networktools (version 1.6.0; Payton Jones; https://cran.r-project.org/package=networktools), mgm (version 1.2.15; Jonas Haslbeck and Lourens J. Waldorp; https://cran.r-project.org/package=mgm), and igraph (version 2.2.1; Gábor Csárdi; https://cran.r-project.org/package=igraph). Only numeric variables were included, and no missing data were present. Three separate network models were estimated from the same dataset using different variable combinations to explore distinct symptom patterns. The Addictions Network comprised nine nodes representing three substance-related (alcohol, tobacco, cannabis) and six behavioral addiction domains. This model was designed to characterize the core structural organization of addictions, identify the strongest interrelationships, and determine which addiction types occupied the most central and/or bridging positions within the Addictions network. The Addictions and Psychometric Features Network included sixteen nodes, integrating addiction variables with clinical and psychosocial constructs (impulsivity, anhedonia, depression, anxiety, stress, chronic stress, and childhood adversity). This model examined the associations between addictions and clinical variables, quantified bridge connections linking behavioral and clinical domains, and assessed whether community structure differentiated addiction and clinical clusters. The Focused Addictions and Psychometric Features Network consisted of fourteen nodes, including alcohol, tobacco, and sexual activity, alongside psychometric features. The focused subnetwork was subsequently constructed as a transdiagnostic, network-informed refinement step to examine the most structurally influential nodes and their immediate cross-domain associations with greater interpretive clarity, rather than as an independent analytic model. This model provided a higher-resolution examination of the addictions most strongly associated with clinical variables, allowing evaluation of which psychological constructs demonstrated the strongest links to these addictions and how these connections influenced centrality, bridging roles, and node predictability (R^2^).

Across all networks, edge-level results with bootstrap confidence intervals, node-level indices (total edge weights, centrality measures, and R^2^), community memberships derived using exploratory graph analysis (EGA) for cluster identification, Walktrap-derived communities used for bridge centrality analyses, and stability indicators (case-dropping Correlation Stability (CS) coefficients and γ-sensitivity analyses) were reported. This multi-model approach enabled the characterization of the addiction core, the identification of clinical–addiction interfaces, and the assessment of the robustness of findings across varying model scopes.

Network Estimation and Visualization:Networks were estimated using the Extended Bayesian Information Criterion Graphical Least Absolute Shrinkage and Selection Operator (EBICglasso) via the estimateNetwork() function in bootnet package in R [26]. After comparing models with gamma (γ) values ranging from 0.00 to 1.00, γ was set at 0.25. This value was selected to provide an optimal balance between network sparsity and interpretability, while preserving stable and theoretically coherent network structures, consistent with prior methodological recommendations. Correlations were calculated using cor_auto, which estimates polychoric and polyserial correlations [30]. Nodes represented variables, and edges represented partial correlations conditioned on all other variables. Visualization employed the Fruchterman-Reingold algorithm, with blue and red edges indicating positive and negative associations, respectively. The Gaussian Graphical Model (GGM) framework was used, as it is suitable for ordinal data and enhances interpretability through regularization [26, 29, 30, 31].

Network Structure: Centrality measures, including strength, expected influence, betweenness, and closeness, were computed. Strength and expected influence were prioritized due to their higher stability, whereas betweenness and closeness were interpreted cautiously [32, 33]. Network stability was assessed using case-dropping CS coefficients, with values >0.50 considered acceptable [26, 33]. Bridge centrality indices were additionally calculated to identify nodes connecting distinct communities, with community assignments for bridge analyses derived using the Walktrap community detection algorithm [34, 35]. Bridge centrality analyses used Walktrap-derived communities, computed on the absolute-weight adjacency matrix, as the community assignment input.

Accuracy and Stability: Non-parametric bootstrapping (7500 iterations) was used to estimate 95% confidence intervals for edge weights, and case-dropping bootstraps evaluated centrality stability [26]. Edges with confidence intervals including zero were interpreted cautiously. CS coefficients were calculated to determine robustness, with values ≥0.50 deemed acceptable and ≥0.70 ideal. Sensitivity analyses varying γ from 0.00 to 1.00 were conducted to examine the impact of regularization on network structure and node rankings [26, 33].

Community Detection and Predictability: EGA was conducted using the EGAnet package to examine dimensional structure and to derive cluster-level community memberships, as well as to visualize EGA-based network representations [27, 28]. In parallel, community memberships used specifically for bridge centrality analyses were identified using the Walktrap community detection algorithm applied to the EBICglasso-estimated networks [35]. Node predictability, reflecting the proportion of variance explained by neighboring nodes, was calculated using the mgm package and displayed as bar plots to enhance interpretability [26].

Network Modeling Strategy: Three complementary EBICglasso networks (γ = 0.25) were estimated to investigate the internal organization of addictive behaviors and their relationships with clinical variables. Each network was characterized using identical analytic metrics, including network structure, edge weights, node centrality indices, bridge centrality, R^2^, and stability and accuracy measures. EGA-derived community structure was used to describe and visualize clustering patterns. EGA was used to derive and visualize community structure, whereas all inferential network metrics were computed on EBICglasso-estimated networks.

## 3. Results

### 3.1 Socio-demographic Characteristics

**Fig. 1.**
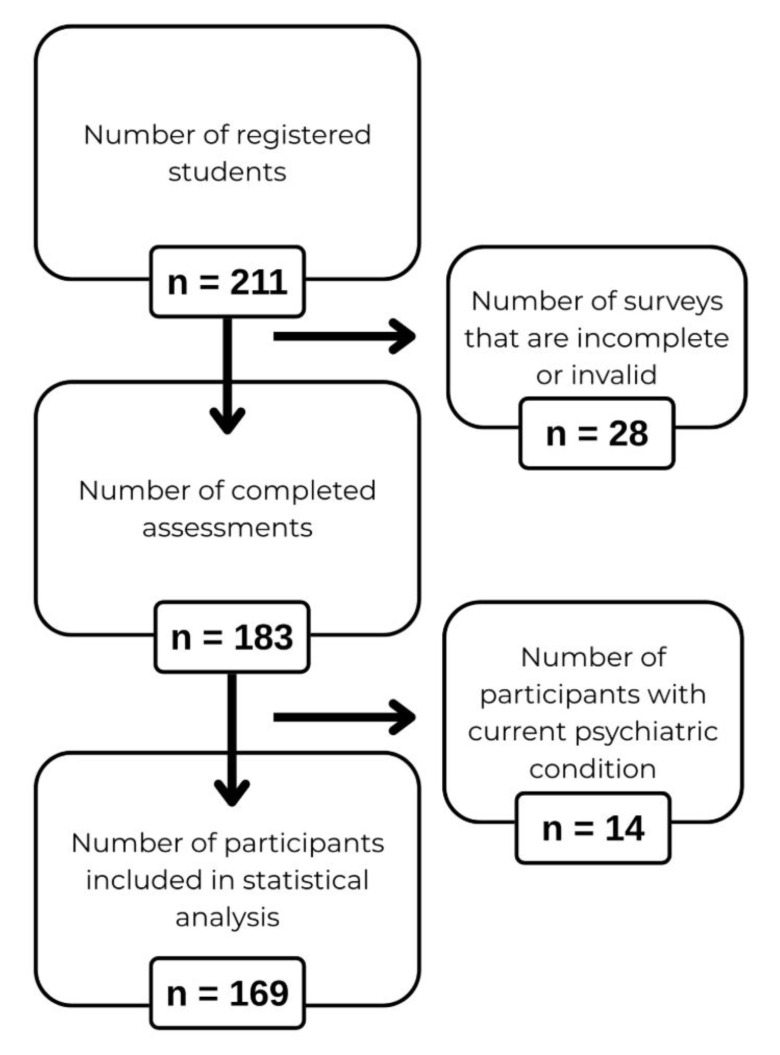
**Flowchart of participant recruitment and inclusion**.

Among the 211 registered students, 183 completed the assessments, corresponding to a completion rate of 86.7%. 28 surveys were excluded due to incomplete or invalid responses, and an additional 14 participants with a current psychiatric diagnosis were excluded. The final analytic sample, therefore, comprised 169 participants (Fig. 1). All subsequent statistical analyses were conducted on this cohort.

The sample consisted of 72.8% females (n = 123) and 27.2% males (n = 46), with a mean age of 20.98 years (SD = 1.7). All participants were undergraduate or graduate students. All were single, while 47.9% reported being in a relationship (Table 1). Most reported no physical illness (95.9%), and 90.5% had no lifetime psychiatric diagnosis. A family history of physical illness was reported by 16.0% and psychiatric disorders by 13.6%.

**Table 1.** **Socio-demographic characteristics of the sample (n = 169)**.

Socio-demographic characteristics		Mean (SD) or n (%)
Age		20.98 (1.7)
Gender		
	Female	123 (72.8)
	Male	46 (27.2)
Marital Status		
	Non-married	169 (100)
	In a relationship	81 (47.9)
	No relationship	88 (52.1)
Employment		
	Unemployed	165 (97.6)
	Part-time worker	4 (2.4)
Education		
	Undergraduate student	144 (85.2)
	Graduate student	25 (14.8)
Income (monthly)		
	None	69 (40.8)
	Below half of the minimum wage	18 (10.7)
	Half of the minimum wage	41(24.3)
	Above the minimum wage	41 (24.3)

SD, Standard Deviation. The average minimum wage was decided at 20,000 Turkish lira (1 USD was 38 Turkish Liras).

Mann-Whitney U tests were used to examine gender differences in psychopathology and substance and behavioral addiction levels. Significant gender differences were observed for anhedonia (U = 2078.00, Z = –2.727, *p* = 0.006) and SSBA domains including gambling (U = 2060.00, *p* < 0.001), shopping (U = 2095.00, *p* = 0.009), gaming (U = 1476.50, *p* < 0.001), and sexual activity (U = 1205.50, *p* < 0.001). No significant gender differences were found for BIS-11, DASS-21 subscales, CSS, or CTQ-33 scores (*p* > 0.05) (**Supplementary Table 1**).

### 3.2 Intercorrelations Among Substance and Behavioral Addictions

The Spearman’s rho correlation analysis was conducted to examine associations among the ten SSBA subscales. Significant positive correlations were observed across multiple addiction types, indicating clustering of addictive behaviors within individuals (Table 2). Alcohol use showed strong associations with tobacco, cannabis, gambling, and sexual behavior (all *p* < 0.001). Tobacco was significantly correlated with cannabis and sexual behavior, while cannabis was also associated with sexual behavior. Gambling demonstrated moderate correlations with gaming, overeating, and sexual behavior. Additional associations emerged between gaming and overeating, and between shopping and overeating. Overall, substance-related addictions were strongly interconnected and also linked to several behavioral addictions.

**Table 2.** **Correlations between addiction domains**.

SSBA domains		1	2	3	4	5	6	7	8	9
Alcohol	r	1.000								
	*p*									
Tobacco	r	0.516^**^	1.000							
	*p*	<0.001								
Cannabis	r	0.426^**^	0.386^**^	1.000						
	*p*	<0.001	<0.001							
Gambling	r	0.296^**^	0.216^**^	0.235^**^	0.202^*^	1.000				
	*p*	<0.001	0.005	0.002	0.009					
Shopping	r	0.105	0.079	0.008	0.018	–0.018	1.000			
	*p*	0.175	0.306	0.918	0.814	0.815				
Gaming	r	0.089	0.092	0.104	0.101	0.270^**^	0.199^*^	1.000		
	*p*	0.249	0.233	0.182	0.195	<0.001	0.009			
Overeating	r	0.094	0.124	0.143	–0.012	0.011	0.319^**^	0.128	1.000	
	*p*	0.223	0.109	0.065	0.877	0.892	<0.001	0.097		
Sexual activity	r	0.429^**^	0.377^**^	0.296^**^	0.002	0.283^**^	0.067	0.313^**^	0.279^**^	1.000
	*p*	<0.001	<0.001	<0.001	0.981	<0.001	0.399	<0.001	<0.001	
Working	r	0.023	–0.127	–0.074	–0.006	–0.175^*^	0.027	0.052	0.071	0.159^*^
	*p*	0.762	0.099	0.345	0.935	0.023	0.728	0.500	0.358	0.043

SSBA, The Brief Screener for Substance and Behavioral Addictions; r, Spearman’s correlation. *p*-values were adjusted using the Bonferroni correction. **p < 0.005 significant; 0.005 ≤ *p < 0.05 marginal.

### 3.3 Impulsivity and Addictive Behaviors

Alcohol and tobacco addictions were significantly associated with all BIS-11 subscales (all *p* < 0.001). Cannabis use correlated with non-planning impulsivity and BIS-11 total scores, while sexual behavior and gambling addictions were linked to multiple impulsivity dimensions. The strongest association was observed between shopping addiction and BIS-11 motor impulsivity. In contrast, working addiction showed moderate negative correlations with impulsivity dimensions, suggesting a distinct behavioral profile (**Supplementary Table 2**).

### 3.4 Depression, Anxiety, and Anhedonia

Alcohol, tobacco, shopping, overeating, sexual behavior, and gaming addictions were positively associated with depressive and anxiety symptoms. Cannabis, gambling, and working addiction showed no significant associations. Anhedonia was selectively related to alcohol and sexual behavior addictions only (**Supplementary Table 3**).

### 3.5 Stress Exposure and Early Life Adversity

Perceived stress was associated with alcohol, shopping, sexual behavior, working, and overeating addictions. Chronic social stress domains (particularly loneliness, partner, financial, workload, and total stress) showed broader associations, with working addiction demonstrating the most extensive pattern (**Supplementary Table 4**). Childhood adversity analyses revealed specific links, notably between sexual behavior addiction and sexual abuse, and cannabis use and emotional abuse, while no associations emerged for gambling, shopping, gaming, or overeating (**Supplementary Table 5**).

### 3.6 Network Analysis Results

#### 3.6.1 The Addictions Network 

**Fig. 2.**
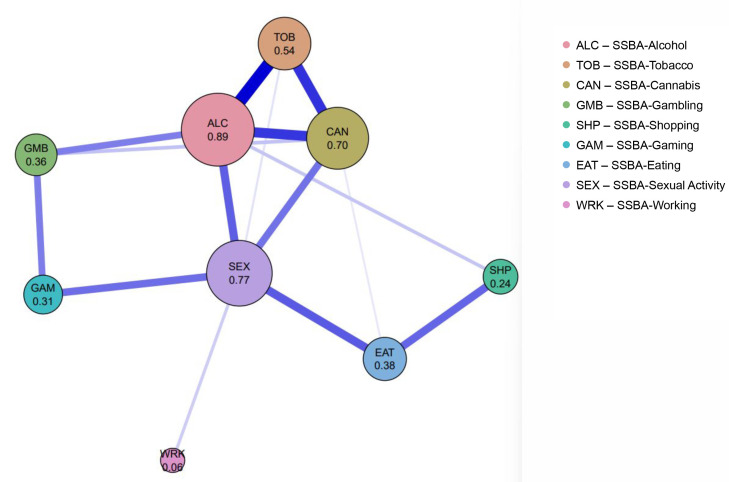
**The addictions network**. Network structure of the nine SSBA addiction domains. Node size and numeric values represent strength centrality, defined as the sum of absolute edge weights. Edge thickness reflects the magnitude of partial correlations. Blue edges indicate positive associations, and red edges indicate negative associations. SSBA, The Brief Screener for Substance and Behavioral Addictions.

The Addictions Network was estimated using the EBICglasso algorithm (γ = 0.25), selected for optimal balance between sparsity and interpretability. The final model included nine addiction-related nodes (alcohol, tobacco, cannabis, gambling, shopping, gaming, eating, sexual activity, and work); cocaine was excluded due to low endorsement. The network retained 15 non-zero edges, most of which were positive, indicating moderate co-variation among addiction domains (Fig. 2 and **Supplementary Table 6**). The strongest and most stable association was tobacco–alcohol, with cannabis showing secondary links to both. Behavioral addictions formed weaker and less consistent connections, and working remained largely isolated.

**Fig. 3.**
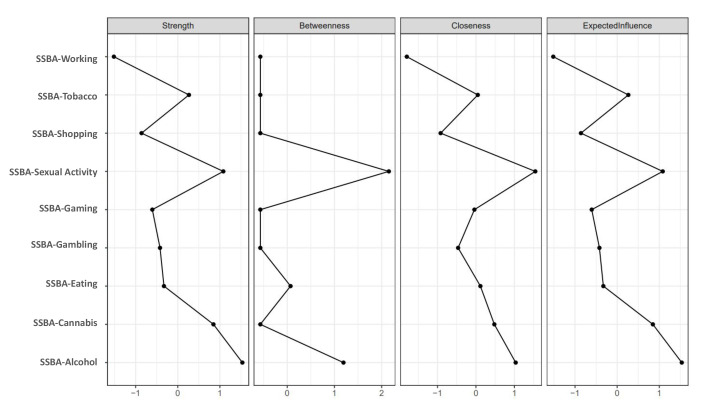
**Centrality indices of the addictions network**. Centrality indices of the nine SSBA addiction domains estimated from the partial correlation network. Panels display strength, betweenness, closeness, and expected influence.

**Fig. 4.**
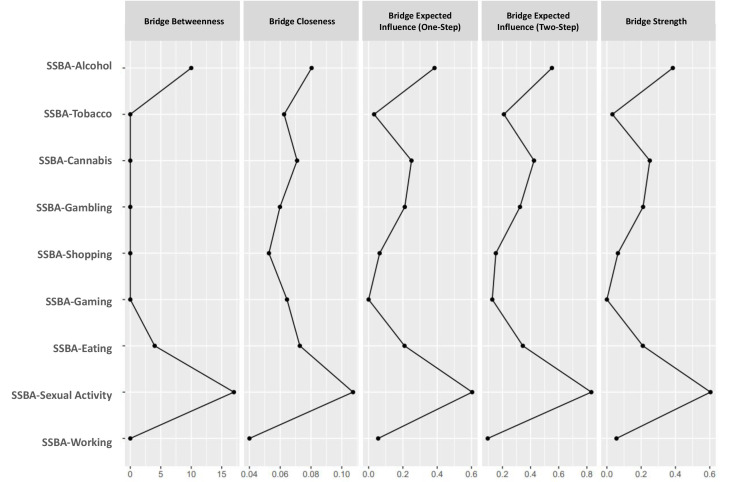
**Bridge centrality indices of the addictions network**. Bridge centrality indices of the SSBA addiction domains are estimated from the partial correlation network. Panels display bridge betweenness, bridge closeness, bridge expected influence (one-step and two-step), and bridge strength.

Centrality indices identified alcohol, sexual activity, cannabis, and tobacco as the most influential nodes, whereas working showed minimal connectivity. Sexual activity and alcohol demonstrated the highest betweenness values, functioning as key intermediaries within the network (Fig. 3 and **Supplementary Table 7**). Bridge centrality further highlighted sexual activity and alcohol as primary connectors between substance and behavioral addictions (Fig. 4 and **Supplementary Table 8**). EGA-based visualization revealed three clusters: substance addictions, a gambling-gaming-sexual activity cluster, and a shopping-eating cluster, with working remaining unclustered (**Supplementary Fig. 1**). Predictability values were highest for alcohol, tobacco, and sexual activity, indicating that these behaviors were most strongly determined by surrounding network structure (**Supplementary Fig. 2**). Stability and sensitivity analyses supported the consistency of the network structure (**Supplementary Table 9**). All results from the Addictions Network analysis are presented in the **Supplementary Material**.

#### 3.6.2 The Addictions and Psychometric Features Network

**Fig. 5.**
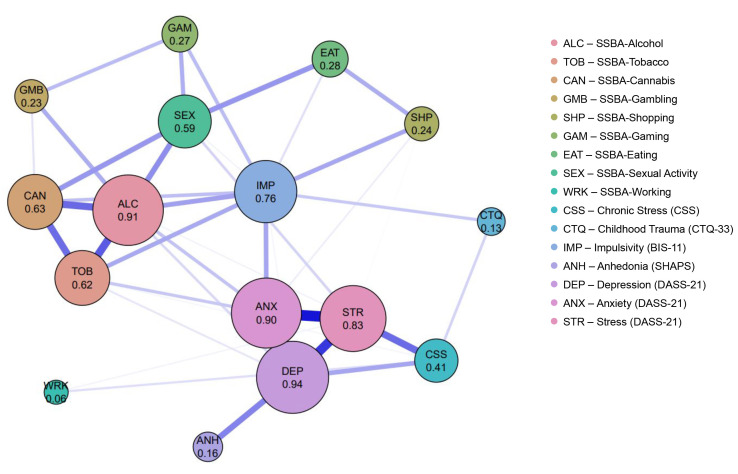
**The addictions and psychometric features network**. Network structure of the nine SSBA addiction domains and associated psychometric features. Node size and numeric values represent strength centrality, defined as the sum of absolute edge weights. Edge thickness reflects the magnitude of partial correlations. Blue edges indicate positive associations, and red edges indicate negative associations. BIS-11, Barratt Impulsiveness Scale-11; CTQ-33, Childhood Trauma Questionnaire-33; CSS, Chronic Stress Scale; DASS-21, Depression Anxiety Stress Scales-21; SHAPS, Snaith-Hamilton Pleasure Scale.

**Fig. 6.**
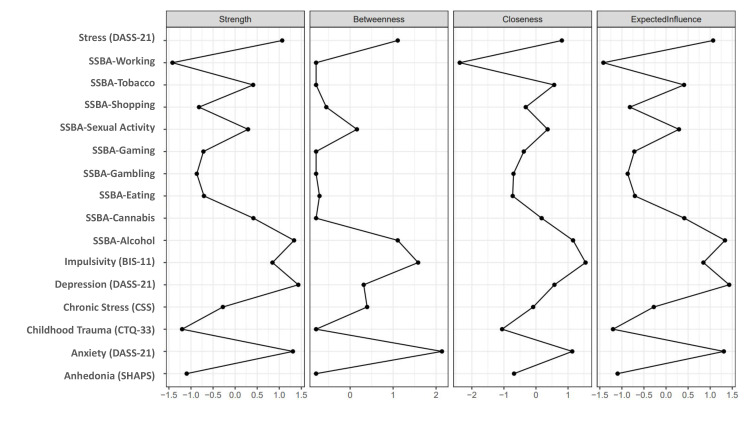
**Centrality indices of the addictions and psychometric features network**. Centrality indices of the extended network integrating SSBA addiction domains and psychometric features. Panels display strength, betweenness, closeness, and expected influence estimated from the partial correlation network.

The network showed two major positively interconnected subnetworks: a substance/behavioral addiction cluster and a clinical-affective distress cluster. In summary, using the EBICglasso model (γ = 0.25), the final network contained 40 positive edges across 16 nodes (Fig. 5), reflecting a moderately dense configuration in which most constructs were positively interconnected (**Supplementary Table 10**). The strongest positive associations emerged within the substance-use core, consisting of alcohol-tobacco, alcohol-cannabis, and cannabis-tobacco (**Supplementary Table 10**). These edges demonstrated high weights and narrow bootstrap confidence intervals, indicating robust co-activation among substance-related behaviors. Within behavioral addictions, meaningful positive connections were observed between eating-sexual activity, eating-shopping, gaming-sexual activity, and gaming-gambling, suggesting a secondary cluster reflecting impulse-driven behavioral tendencies. The clinical subnetwork exhibited the strongest edges in the entire model, particularly the depression-anxiety, anxiety-stress, and depression-stress links. Centrality analyses identified depression, alcohol, anxiety, stress, and impulsivity as the most influential nodes, each showing high strength, expected influence, and/or betweenness (Fig. 6 and **Supplementary Table 11**). In contrast, working addiction remained peripheral, with very weak and inconsistent connections. Cross-domain edges identified impulsivity and anxiety as key bridge nodes linking the addiction and clinical clusters (**Supplementary Fig. 3** and **Supplementary Table 12**). The strongest cross-domain connection was anxiety-impulsivity, followed by alcohol-impulsivity and sexual activity-anxiety, positioning these variables as key transmission points through which affective distress and addiction-related processes interact. Anhedonia displayed a moderate positive association with depression, consistent with partial overlap between these constructs. The EGA result confirmed a stable two-cluster solution, separating addiction-related constructs from clinical-psychological variables, with limited but meaningful cross-cluster positive associations (**Supplementary Fig. 4**). Across all nodes, depression (R^2^ = 0.33), anxiety (R^2^ = 0.30), and stress (R^2^ = 0.30) exhibited the highest predictability values, indicating that a substantial proportion of their variance was explained by their connections to other variables in the network. Impulsivity (R^2^ = 0.25), alcohol (R^2^ = 0.22), tobacco (R^2^ = 0.19), and sexual activity (R^2^ = 0.17) also showed moderately high levels of variance explained by their neighboring nodes, suggesting that these constructs were similarly influenced by the broader network structure (**Supplementary Fig. 5**). Stability and sensitivity analyses supported the consistency of the network structure (**Supplementary Table 13**). Overall, the results indicate a stable and interpretable network structure, characterized by strongly interconnected substance use and affective cores linked through impulsivity and anxiety.

#### 3.6.3 The Focused Addictions and Psychometric Features Network 

The Focused Addictions and Psychometric Features Network, integrating affective symptoms, impulsivity, stress domains, childhood trauma, and selected addictive behaviors (alcohol, tobacco, and sexual activity), showed a coherent and highly interconnected structure dominated by positive associations. Using the EBICglasso model (γ = 0.25), a 14-node network with 50 non-zero edges was obtained, indicating considerable cross-domain coupling (**Supplementary Fig. 6** and **Supplementary Table 14**). The strongest edges were observed between anxiety-depression, stress-anxiety, and alcohol-tobacco, reflecting a closely interconnected affective distress core and a robust substance-use dyad. Additional strong positive relations included stress-depression, alcohol-sexual activity, and emotional abuse-sexual abuse, underscoring the internal coherence of both the affective and trauma subnetworks. Moderately strong edges such as depression-anhedonia, stress-loneliness stress, and impulsivity-alcohol further linked mood, stress, social isolation, and addictive behaviors. Centrality indices showed that anxiety, depression, and stress had the highest strength and expected influence, followed closely by alcohol, loneliness stress, tobacco, and impulsivity (**Supplementary Fig. 7** and **Supplementary Table 15**). This pattern indicates that affective symptoms and alcohol use constitute the main hubs of the system, with tobacco, impulsivity, and loneliness acting as secondary but influential connectors. Betweenness centrality highlighted emotional abuse, loneliness stress, depression, and anxiety as nodes with higher values among domains. Bridge centrality analyses identified loneliness stress, anxiety, stress, impulsivity, tobacco, alcohol, and emotional abuse as major cross-cluster connectors (**Supplementary Fig. 8** and **Supplementary Table 16**). These nodes jointly integrate addiction, stress, and trauma clusters, suggesting plausible pathways from early adversity and interpersonal stress to affective dysregulation and addictive behaviors. The EGA revealed a stable three-cluster solution comprising: (i) an addiction-impulsivity cluster (alcohol, tobacco, sexual activity, impulsivity), (ii) a stress-affective cluster (financial, partner, loneliness stress, depression, anxiety, stress, anhedonia), and (iii) a trauma cluster (emotional and sexual abuse) (**Supplementary Fig. 9**). Predictability was highest for depression, anxiety, and stress, and moderate for alcohol, impulsivity, tobacco, and loneliness stress, indicating that these nodes are strongly determined by their network context (**Supplementary Fig. 10**). Stability indices (CS = 0.52 for strength and expected influence) and γ-sensitivity analyses supported the consistency and stability of the network structure (**Supplementary Table 17**).

## 4. Discussion

This study suggests that substance-related and behavioral addictions are organized in an interconnected structural system in which alcohol, tobacco, and sexual activity appear as relatively central nodes in this non-clinical sample. Impulsivity and anxiety emerged as prominent cross-domain interface variables linking addictive behaviors with internalizing symptoms. The affective-distress cluster, comprising depression, anxiety, and stress, was characterized by relatively strong interconnections and higher predictability within the Addictions and Psychometric Features Network. Overall, these findings are consistent with the notion of shared vulnerability patterns, in which impulsivity and anxiety-related constructs may be associated with the co-occurrence of addictions and internalizing disorders. Accordingly, given the cross-sectional design and the non-clinical nature of the sample, the present findings should be interpreted as descriptive and exploratory, and their clinical applicability requires replication in diagnosed and more heterogeneous samples.

Our findings indicated clustering patterns among substance-related and behavioral addictions, with alcohol, tobacco, and cannabis showing relatively stronger interconnections, while sexual activity, gaming, gambling, shopping, and overeating showed structured patterns of co-variation. This pattern is broadly consistent with previous literature suggesting that addictive behaviors often co-occur rather than presenting in isolation. For example, Burleigh *et al.* (2019) [36] reported that gaming disorder frequently co-occurs with substance use, internet, gambling, and pornography-related problems, supporting our observation that gaming was linked not only to gambling but also to overeating and sexual activity. Similarly, a large epidemiological clustering study by Konkolÿ Thege *et al*. (2016) [1] identified distinct addiction profiles (tobacco, overeating, shopping, sexual activity, gaming, working, and poly-addiction) consistent with our differentiated yet interconnected clusters. Taken together, these findings suggest that conceptualizing addictive behaviors as interconnected phenomena may be informative for future research and prevention-oriented frameworks, rather than viewing them solely as isolated behaviors.

The clustering pattern observed in the Addictions Network, particularly among gambling, gaming, sexual activity, shopping, and overeating is broadly consistent with dimensional models of behavioral addictions. The moderate associations between gambling, gaming, sexual activity, and overeating in our sample are comparable to network-analytic findings by Somma *et al*. (2023) [37], who described that gambling aligns with an addiction/impulsivity dimension, whereas compulsive buying aligns more closely with compulsivity. In a similar manner, shopping and overeating in the present network were more closely associated with each other, whereas gambling, gaming, and sexual activity showed relatively stronger associations with impulsivity-related constructs. Related clinical literature has reported that compulsive buying and gambling frequently co-occur and are associated with greater clinical severity [38], and that compulsive sexual behaviors often co-occur with gambling and substance use, with impulsivity and emotion-regulation deficits [39]. These findings may indicate contextual support rather than direct clinical generalization.

Our finding that sexual activity occupied a relatively central position across domains is broadly consistent with prior reports that sexual compulsivity frequently co-occurs with gambling and pornography use [40]. Sussman *et al.* (2015) [41] reported high level co-occurrence across 22 addiction types in emerging adults, while Schou Andreassen *et al*. (2016) [42] demonstrated overlapping, but distinct, profiles for social media and gaming addiction, with variations associated with demographic and psychiatric factors. In addition, the observed overlap between shopping, gaming, gambling, and internet-related behaviors is comparable to previous findings reporting associations between problematic social media use and other behavioral addictions [43].

In the Addictions Network, centrality analyses indicated alcohol (0.89), sexual activity (0.77), and cannabis (0.70) as the nodes with the highest total connections in the network. This pattern suggests that these nodes occupy relatively central positions within the network structure, reflecting a higher degree of connectivity compared to other addiction domains.

Specifically, sexual activity showed the highest bridge strength value (0.60) indicating a relatively strong structural connection between substance addictions (alcohol, cannabis) and behavioral addictions (gambling, gaming). This observation is consistent with the literature reporting associations between sexual addiction, dysregulation and impulsivity, which have been discussed in transdiagnostic frameworks [44]. In contrast, working addiction showed relatively low connectivity and was positioned at the periphery of the Addictions Network, despite exhibiting moderate negative bivariate correlations with impulsivity dimensions. This difference reflects the distinction between bivariate associations and partial correlations estimated in the EBICglasso network, in which shared variance with other addiction domains is controlled and impulsivity was not included as a node.

Our findings show that all addictive behaviors except working addiction were positively associated with impulsivity, suggesting that impulsivity may be a common correlate across different addiction domains in this non-clinical sample. Impulsivity has been widely discussed in relation to substance use disorders, and in the present network it showed relatively stronger associations with tobacco and alcohol use, which is broadly consistent with prior literature reporting links between impulsive traits and nicotine- and alcohol-related problems [8, 45]. Cannabis use in the current sample was also associated with impulsivity, in line with previous reports describing elevated impulsivity among individuals reporting problematic cannabis use [46]. Beyond substances, gambling, shopping, gaming, and sexual behaviors also showed positive associations with impulsivity. This pattern is broadly consistent with meta-analytic evidence showing that individuals with gambling disorder display elevated impulsivity across multiple domains, including motor impulsivity, impaired response inhibition, and steeper delay discounting [47]. Similarly, previous studies have reported associations between inhibitory control difficulties, impulsivity, and problematic internet use among medical students [48]. Notably, alcohol, tobacco, cannabis, and sexual activity correlated with all impulsivity subdimensions, highlighting the multidimensional nature of impulsivity in relation to addiction-related symptom patterns.

Our findings showed that anhedonia was associated with sexual behavior and alcohol addiction, while no such associations were observed with other addictive behaviors in this sample. This selective pattern is consistent with prior evidence that suggesting that associations between anhedonia and addictive behaviors vary across addiction types rather than occurring uniformly. For alcohol use, this finding is comparable to results reported by Stamatovich *et al*. (2025) [49], who described associations between specific forms of anhedonia, particularly social anhedonia, and problematic drinking, alongside impulsivity-related responses to negative social expectations. Large epidemiological studies have similarly reported that anhedonia is more frequently observed in moderate-to-severe alcohol use disorder, but not milder forms of alcohol use [50]. The link between anhedonia and sexual behavior is comparable to prior studies reporting links between anhedonia and addictive tendencies in internet use and gaming contexts, potentially reflecting broader alterations in reward sensitivity [51, 52]. In contrast, the absence of associations with cannabis, gaming, shopping, or gambling in our dataset is consistent with reports showing that anhedonia does not uniformly increase addiction risk but may appear selectively in anticipatory rather than consummatory aspects of reward processing [43]. It is also consistent with longitudinal findings indicating that anhedonia becomes a significant predictor of internet-related addictive behaviors only when examined prospectively over time [53]. Overall, these results point to a nuanced pattern in which anhedonia shows selective associations with certain addictive behaviors, potentially depending on reward-processing profiles and behavioral context.

Our results indicated that childhood adversity was selectively associated with several addictions (most strongly with sexual behavior and cannabis) while no clear associations were observed with gambling, shopping, gaming, and overeating. These patterns are broadly consistent with prior evidence linking that trauma-related affective distress to specific substance-related behavior patterns. For example, a prior clinical study reported that individuals with comorbid substance use disorder and post-traumatic stress disorder present with elevated negative affect, perseverative thinking, and distress intolerance [6]. These observations may provide contextual support for the associations observed between indicators of sexual or physical abuse and addiction-related symptom patterns in this non-clinical sample. Stress-related associations also showed specificity, with perceived stress was associated with alcohol use, shopping, sexual activity, working, and overeating, while chronic stress domains showed broader associations across behavioral addictions. This pattern is comparable to previous findings reporting associations between compulsive, and stress, anxiety, depression, and impulsivity [7]. Additionally, lifetime and early-life stress have been linked to impulsivity and addictive behaviors, particularly those associated with food addiction [9]. Longitudinal evidence has suggested that cumulative lifetime stress predicts both impulsivity and a heightened vulnerability to addictive behaviors [9]. These results point to trauma- and stress-related patterns of association across different addictive behaviors.

In the Addictions and Psychometric Features Network, the strong clustering of impulsivity with behavioral addictions within the addiction cluster is consistent with theoretical models that conceptualize impulsivity is a central vulnerability-related construct in addictive behaviors.

Impulsivity was identified as a node with high centrality (0.76) and as the second strongest bridge node after anxiety (bridge strength = 0.20, bridge betweenness = 21). This pattern suggests that impulsivity is positioned at the interface between emotional distress (anxiety, depression) and addictive behaviors. The positive relationship between impulsivity and alcohol use (0.166) is consistent with findings showing that impulsivity subscales, particularly urgency and sensation seeking, predict alcohol misuse among young adults [49]. The relation of impulsivity with gaming, gambling, and eating addictions in the Addictions and Psychometric Features Network are consistent with an extensive literature showing that impulsivity as a transdiagnostic feature across various behavioral addictions including gambling disorder, gaming addiction, and eating addiction. Recent evidence further indicates that under conditions of heightened stress, such as during the COVID-19 pandemic, emotion-driven impulsivity significantly predicted increases in addictive behavior severity [54].

The position of anhedonia in the Focused Addictions and Psychometric Features Network constitutes a notable aspect of the present findings. Anhedonia was positioned within the clinical/psychosocial cluster (C2) alongside other emotional distress variables such as depression, anxiety, and stress. This is consistent with previous evidence indicating that anhedonia, as described in the DSM-5, is a core symptom of depression. Anhedonia exhibited relatively low total connection strength in the network (0.407). Specifically, it showed zero bridge strength and zero betweenness values in relation to the clinical and addiction clusters. This finding suggests that anhedonia may be more closely related to the internal organization of the core emotional distress, rather than in the addiction cluster itself, and that it does not function as a direct bridging feature linking emotional distress to addictive behaviors in this specific student population. Importantly, this pattern should not be interpreted as indicating a lack of relevance of anhedonia in addiction-related psychopathology. Prior evidence indicates that anhedonia is a robust predictor of relapse in alcohol use disorder, with individuals who relapse exhibiting higher levels of anhedonia [50]. However, in this cross-sectional sample of university students, anhedonia can be better represented as a shared vulnerability accompanying addiction comorbidity rather than as an active bridge, as assumed by anxiety and impulsivity. Furthermore, evidence linking anticipatory anhedonia but not consummatory anhedonia in the context of cannabis use highlights the importance of anhedonia subtypes [55]. Accordingly, the use of a single general anhedonia scale (SHAPS) in this study may have limited the differentiation of anhedonia into its anticipatory and consummatory subscales, potentially leading to the loss of nuances. Together, these findings support a multidimensional addiction architecture in which substance and behavioral addictions form interrelated clusters associated with shared mechanisms of impulsivity, compulsivity, and emotional distress.

The Addictions and Psychometric Features Network of this study, depression, anxiety, and stress (the DASS-21 triad) constituted a clearly defined affective distress core by exhibiting the strongest internal connections (w = 0.312, w = 0.297, w = 0.253, respectively) and the highest centrality among the clinical variables.

Within this core, anxiety was not only highly central (0.90) but also identified as the strongest bridge node connecting the addiction cluster to the clinical cluster (bridge strength = 0.29, D = 35). This finding supports theoretical models proposing that emotional avoidance or negative affect-based coping strategies are relevant to the association with addiction behaviors. Recent evidence further demonstrates that impulsivity significantly moderates the association between anxiety and problem gambling severity, such that higher anxiety is associated with greater gambling-related harm among individuals with elevated impulsivity [56].

The Focused Addictions and Psychometric Features Network, which integrated stress domains, trauma exposure, impulsivity, internalizing symptoms, and selected addictive behaviors displayed a network structure in which stress-related and trauma-related variables occupied structurally central positions. Loneliness stress, partner stress, emotional abuse, and sexual abuse demonstrated high bridge centrality, suggesting that chronic interpersonal strain and early-life adversity may be positioned at the interface between affective distress and addictive behaviors within the network. This structural pattern is consistent with prior evidence reporting associations between chronic social stressors, elevated negative affectivity, and greater engagement in maladaptive coping behaviors, including compulsive shopping, overeating, and alcohol use [7, 9]. The clustering of emotional abuse and sexual abuse within the trauma subnetwork and their proximity to impulsivity is consistent with theoretical models proposing associations between early adversity, alterations in inhibitory control and stress-reward processing, which have been linked to both internalizing symptoms and addiction-related outcomes [6]. The prominent role of anxiety and stress as bridge nodes further suggests that affective hyperarousal may be relevant to the associations observed between trauma exposure to addictive behavior, a pattern observed across both clinical and community samples. These findings are consistent with a transdiagnostic framework in which early adversity, chronic stress, and affective distress co-occur and are jointly associated with a range of addiction-related outcomes.

Across the addiction-only, extended, and focused network models, a convergent structural pattern emerged. Substance-related addictions consistently formed a central core, affective distress variables clustered tightly, and impulsivity and anxiety repeatedly occupied interface positions linking addictive behaviors with internalizing symptoms, underscoring coherence across analytic levels. This convergence across models suggests that comorbidity among substance-related and behavioral addictions may reflect shared vulnerability processes operating at a network-structural level rather than disorder-specific mechanisms. Importantly, these observations are descriptive and exploratory, and should be interpreted in line with the cross-sectional and non-clinical nature of the sample.

### 4.1 Limitations

This study has several limitations that should be considered when interpreting the findings. The cross-sectional design prevents the establishment of causal inferences and limits conclusions regarding temporal relationships among stress, early adversity, impulsivity, and addiction vulnerability. The sample consisted of non-clinical university students, which reduces clinical heterogeneity and may limit generalizability to broader or clinical populations with more severe addiction profiles. Accordingly, addiction-related measures likely reflect subclinical or normative engagement, and restricted variance may have influenced the estimated network structure as well as the interpretation of centrality and bridge metrics. For this reason, the present findings should be interpreted as descriptive and hypothesis-generating within a non-clinical Turkish university student sample and may not generalize to older, clinically diagnosed, or cross-cultural populations.

In addition, the low prevalence of cocaine use restricted conclusions regarding stimulant-related behaviors. All data were collected using self-report measures, which are subject to recall bias, social desirability effects, and shared method variance. Although network analytic techniques provide nuanced insights into complex associations, the modest sample size may have limited the stability and precision of some estimated edges and centrality metrics, as reflected by wide bootstrap confidence intervals and moderate stability coefficients, indicating that larger samples are needed to improve the robustness and reliability of network estimates. Furthermore, the imbalanced sex distribution of the sample (predominantly female) may have influenced the observed network structure, and potential sex-specific network differences could not be examined.

Finally, childhood adversity was included as a theory-driven indicator of early-life stress rather than as a comprehensive assessment of all environmental risk factors relevant to addiction vulnerability. Childhood trauma was assessed retrospectively, and the absence of objective or multi-informant measures may have influenced the precision of trauma–addiction associations.

### 4.2 Strengths

Despite these limitations, this study has several notable strengths. It represents one of the few investigations integrating substance and behavioral addictions with impulsivity, anhedonia, internalizing symptoms, early adversity, and chronic stress in a unified network model. The multidimensional assessment allowed identification of distinct addiction clusters and high-impact bridge nodes, particularly alcohol, sexual activity, anxiety, and impulsivity, offering a detailed understanding of shared vulnerability structures. The use of validated Turkish versions of all scales, rigorous statistical quality control (including attention checks), and advanced network modeling (EBICglasso, EGA, bootstrapping) enhanced internal validity and analytic precision. Examining both perceived stress and domain-specific chronic stressors provided a nuanced view of stress-addiction relationships rarely incorporated in similar studies.

### 4.3 Future Perspectives

Future research should incorporate longitudinal designs to clarify developmental trajectories linking stress exposure, trauma, and impulsivity to addiction onset and maintenance. Expanding the sample to include clinical populations, balanced sex distribution, diverse age groups, and individuals with diagnosed substance use disorders will improve generalizability and allow examination of more severe addiction phenotypes. Multi-method assessments, such as behavioral impulsivity tasks, neurobiological markers, and ecological momentary assessments, could complement self-report data and capture dynamic processes underlying addictive behaviors. Integrating neuroimaging, genetic, or inflammatory markers may further illuminate mechanistic pathways linking internalizing psychopathology with addiction risk. Finally, identifying modifiable bridge nodes suggests promising intervention targets; future trials could test whether reducing impulsivity or affective distress disrupts cross-domain addiction clustering, supporting personalized prevention and treatment approaches.

## 5. Conclusion

In conclusion, this study suggests that substance-related and behavioral addictions can be conceptualized as an integrated and multidimensional network characterized by shared vulnerability-related features. Substance-use behaviors, particularly alcohol, tobacco, and cannabis, clustered into a central core, while sexual activity emerged as a structurally influential position connecting substance-related and behavioral addictions. Impulsivity and anxiety were identified as prominent transdiagnostic features positioned between clusters with internalizing symptoms, while stress and early trauma showed strong associations with affective distress. The consistent clustering patterns, structurally important bridge nodes, and affective-distress core observed across all three networks illustrate the interconnected organization of addictive behaviors and related psychopathology. These findings should be interpreted as exploratory and descriptive, and do not imply causal mechanisms or direct clinical intervention targets. In this context, the results may inform future research by highlighting the potential relevance of impulsivity, emotional distress, and trauma-related factors in understanding cross-domain addictive behaviors; clinically, they should be interpreted as hypothesis-generating, given the non-clinical and cross-sectional nature of the data, and require validation in clinically diagnosed samples.

## Data Availability

The datasets used and analyzed during the current study are available from the corresponding authors on reasonable request.

## Abbreviations

BIS-11, Barratt Impulsiveness Scale-11; CSS, Chronic Stress Scale; CTQ-33, Childhood Trauma Questionnaire-33; DASS-21, Depression Anxiety Stress Scales-21; DSM-5, Diagnostic and Statistical Manual of Mental Disorders, Fifth Edition; EBICglasso, Extended Bayesian Information Criterion Graphical Least Absolute Shrinkage and Selection Operator; EGA, Exploratory Graph Analysis; GGM, Gaussian Graphical Model; ICD-11, International Classification of Diseases, Eleventh Revision; R^2^, node predictability; SD, standard deviation; SHAPS, Snaith-Hamilton Pleasure Scale; SSBA, The Brief Screener for Substance and Behavioral Addictions.

## Supplementary Material

Supplementary material associated with this article can be found, in the online version, at https://doi.org/10.31083/AP49511.
