# Supplementary material for: Phenotypic Clustering of Addictions Reveals Impulsivity Links to Internalizing Disorders: A Network Analysis
Source: Alpha Psychiatry. 2026 Mar 9;27(2):49511. doi: 10.31083/AP49511 (PMC13156064; doi:10.31083/AP49511)
Supplement: Supplementary file 1 [file 2757-8038-27-2-49511-s1.zip › Supplementary Material.docx]

Supplementary Materials

# 1. Correlation and Comparison Analyses

**Supplementary Table 1. Descriptive statistics and sex-based comparisons for SSBA domains and clinical measures.**

|  |  | | All sample | | | Female | | | Male | | |  |  |
| --- | --- | --- | --- | --- | --- | --- | --- | --- | --- | --- | --- | --- | --- |
| SSBA Domains | N Total | N* | | Range | Mean (SD) | N | Range | Mean (SD) | N | Range | Mean (SD) | Z | p |
| **Alcohol**  **Tobacco**  **Cannabis**  **Gambling**  **Shopping**  **Gaming**  **Eating**  **Sexual activity**  **Working** | 169   169   167   169   169   169   169   163   169 | 134  101  29  40  164  116  165  106  128 | | 0-13  0-16  0-6  0-10  0-16  0-14  0-16  0-15  0-16 | 3.21 (3.18)  3.85 (4.87)  0.41(1.10)  0.59(1.42)  5.83(3.63)  3.01(3.62)  6.20(3.62)  3.17(3.32)  4.12(4.01) | 123  123  121  123  123  123  123  118  123 | 0-13  0-15  0-5  0-10  0-16  0-13  0-16  0-12  0-16 | 3.14 (3.11)  3.86 (4.97)  0.41 (1.12)  0.42 (1.34)  6.28 (3.65)  2.14 (2.93)  6.30 (3.65)  2.31 (2.82)  4.05 (3.97) | 46  46  46  46  46  46  46  45  46 | 0-13  0-16  0-6  0-5  0-12  0-14  1-15  0-15  0-16 | 3.39(3.37)  3.83(4.63)  0.41 (1.09)  1.02 (1.56)  4.65 (3.32)  5.33 (4.23)  5.94 (3.54)  5.44 (3.47)  4.33(4.14) | -0.230  -0.339  -0.410  -3.651  -2.604  -4.880  -0.679  -5.511  -0.337 | 0.818  0.735  0.682  **<0.001**  **0.009**  **<0.001**  0.497  **<0.001**  0.736 |
| Clinical Measures | N Total | | | Range | Mean (SD) | N | Range | Mean (SD) | N | Range | Mean (SD) | Z | p |
| **DASS-21-DEP**  **DASS-21-ANX**  **DASS-21-STR**  **CSS**  **CTQ-33**  **BIS-11**  **SHAPS** | 169  169  169  169  169  169  169 | | | 0-21  0-21  0-20  1-80  25-85  38-91  0-10 | 6.20 (4.24)  5.69 (4.05)  7.92 (4.01)  22.07 (11.28)  36.40 (10.53)  61.60(9.60)  2.01(2.33) | 123123  123  123  123  123  123 | 0-21  0-18  0-19  2-80  25-85  38-88  0-10 | 6.20 (4.24)  5.85 (3.95)  8.07 (3.95)  21.50(11.45)  36.12(10.09)  61.29(9.44)  1.76 (2.24) | 46  46  46  46  46  46  46 | 0-19  0-21  0-20  1-50  25-78  44-91  0-10 | 6.20(4.37)  5.24 (4.31)  7.54 (4.19)  23.59(10.77)  37.13(11.74)  62.44(10.06)  2.67(2.45) | -0.069  -1.416  -0.962  -1.382  -0.081  -0.369  -2.727 | 0.945  0.157  0.336  0.167  0.935  0.712  **0.006** |

BIS-11, Barratt Impulsiveness Scale-11; CTQ-33, Childhood Trauma Questionnaire-33; CSS, Chronic Stress Scale; DASS-21, Depression Anxiety Stress Scales-21; SHAPS, Snaith-Hamilton Pleasure Scale; SSBA, The Brief Screener for Substance and Behavioral Addictions; N, Number; SD, Standard Deviation; Z, Mann-Whitney U Test.

Statistically significant associations (p < 0.05) are shown in bold.

* SSBA ≥ 1, participants who had indicated they had engaged in the target behavior in the past 12 months.

**Supplementary Table 2. Correlations of SSBA domains with impulsivity subscales and total score.**

| SSBA Domains |  | Non-Planning | Motor | Attention | BIS-11 Total |
| --- | --- | --- | --- | --- | --- |
| **Alcohol** | r | 0.334** | 0.363** | 0.328** | 0.391** |
|  | p | <0.001 | <0.001 | <0.001 | <0.001 |
| **Tobacco** | r | 0.283** | 0.283** | 0.328** | 0.345** |
|  | p | <0.001 | <0.001 | <0.001 | <0.001 |
| **Cannabis** | r | 0.260** | 0.170* | 0.214* | 0.263** |
|  | p | <0.001 | 0.028 | 0.006 | <0.001 |
| **Gambling** | r | 0.272** | 0.186* | 0.104 | 0.254** |
|  | p | <0.001 | 0.015 | 0.178 | <0.001 |
| **Shopping** | r | 0.161* | 0.407** | 0.208* | 0.271** |
|  | p | 0.036 | <0.001 | 0.007 | <0.001 |
| **Gaming** | r | 0.193* | 0.142 | 0.089 | 0.178* |
|  | p | 0.012 | 0.066 | 0.252 | 0.021 |
| **Overeating** | r | 0.149 | 0.14 | 0.150 | 0.187* |
|  | p | 0.053 | 0.069 | 0.051 | 0.015 |
| **Sexual activity** | r | 0.270** | 0.269** | 0.185* | 0.295** |
|  | p | <0.001 | <0.001 | 0.018 | <0.001 |
| **Working** | r | -0.180* | 0.005 | -0.202* | -0.180* |
|  | p | 0.019 | 0.951 | 0.008 | 0.019 |

SSBA, The Brief Screener for Substance and Behavioral Addictions; BIS-11, Barratt Impulsivity Scale

r: Spearman’s correlation. p-values were adjusted using the Bonferroni correction.
**p < 0.005 significant; 0.005 ≤ *p < 0.05 marginal

**Supplementary Table 3. Correlations between SSBA domains and depressive symptoms, anxiety, stress, and anhedonia.**

| SSBA Domains |  | DASS-21 Depression | DASS-21Anxiety | SHAPS - Anhedonia |
| --- | --- | --- | --- | --- |
| **Alcohol** | r | **0.272**** | **0.303**** | **0.164*** |
|  | p | <0.001 | <0.001 | 0.034 |
| **Tobacco** | r | **0.181*** | **0.266**** | -0.033 |
|  | p | 0.018 | <0.001 | 0.666 |
| **Cannabis** | r | 0.139 | 0.074 | 0.059 |
|  | p | 0.073 | 0.342 | 0.446 |
| **Gambling** | r | -0.031 | -0.024 | 0.079 |
|  | p | 0.692 | 0.761 | 0.305 |
| **Shopping** | r | **0.211*** | **0.241**** | 0.026 |
|  | p | 0.006 | 0.002 | 0.736 |
| **Gaming** | r | **0.153*** | **0.186*** | 0.123 |
|  | p | 0.047 | 0.015 | 0.111 |
| **Overeating** | r | **0.248**** | **0.188*** | 0.089 |
|  | p | 0.001 | 0.014 | 0.252 |
| **Sexual activity** | r | **0.232**** | **0.191*** | **0.214*** |
|  | p | 0.003 | 0.015 | 0.006 |
| **Working** | r | 0.054 | 0.079 | 0.100 |
|  | p | 0.488 | 0.309 | 0.197 |

SSBA, The Brief Screener for Substance and Behavioral Addictions; DASS-21, Depression Anxiety Stress Scales-21; SHAPS, Snaith Hamilton Anhedonia Pleasure Scale; r: Spearman’s correlation.
p-values were adjusted using the Bonferroni correction. Statistically significant associations (**p < 0.005 significant; 0.005 ≤ *p < 0.05 marginal) are shown in bold.

**Supplementary Table 4. Correlations of SSBA domains with total stress and stress dimensions.**

| **SSBA**  **Domains** |  | | **DASS-21**  **Stress** | **CSS**  **Partner** | **CSS**  **Child** | **CSS**  **Work** | **CSS Loneliness** | **CSS**  **Financial** | **CSS**  **Workload** | **CSS**  **Debt** | **CSS**  **Relationship Inoccupancy** | **CSS**  **Family Health** | **CSS**  **Residence** | **CSS**  **Family** | **CSS**  **Ex Partner** | **CSS**  **Others** | **CSS**  **TOTAL** |
| --- | --- | --- | --- | --- | --- | --- | --- | --- | --- | --- | --- | --- | --- | --- | --- | --- | --- |
| **Alcohol** | | r | 0.306** | 0.184* | -0.091 | 0.070 | 0.282** | 0.015 | 0.105 | -0.015 | 0.131 | 0.083 | 0.099 | 0.149 | -0.005 | 0.072 | 0.241** |
|  | | p | <0.001 | 0.016 | 0.237 | 0.363 | <0.001 | 0.844 | 0.175 | 0.851 | 0.089 | 0.282 | 0.200 | 0.054 | 0.953 | 0.354 | 0.002 |
| **Tobacco** | | r | 0.143 | 0.265** | -0.084 | .184* | 0.178* | 0.073 | 0.044 | -0.007 | 0.050 | 0.006 | 0.222** | 0.156* | 0.059 | 0.140 | 0.260** |
|  | | p | 0.063 | <0.001 | 0.278 | 0.017 | 0.020 | 0.347 | 0.570 | 0.929 | 0.520 | 0.937 | 0.004 | 0.043 | 0.448 | 0.070 | <0.001 |
| **Cannabis** | | r | 0.072 | 0.103 | 0.067 | 0.005 | 0.139 | 0.055 | 0.001 | 0.024 | 0.073 | -0.029 | 0.050 | 0.026 | 0.095 | 0.117 | 0.134 |
|  | | p | 0.357 | 0.187 | 0.388 | 0.950 | 0.073 | 0.483 | 0.986 | 0.763 | 0.351 | 0.712 | 0.524 | 0.742 | 0.224 | 0.131 | 0.085 |
| **Gambling** | | r | -0.015 | 0.158* | 0.137 | -0.081 | -0.036 | -0.031 | -0.129 | -0.073 | -0.047 | 0.010 | 0.063 | -0.009 | 0.056 | -0.041 | -0.016 |
|  | | p | 0.848 | 0.040 | 0.075 | 0.294 | 0.641 | 0.690 | 0.094 | 0.344 | 0.542 | 0.893 | 0.414 | 0.908 | 0.472 | 0.597 | 0.834 |
| **Shopping** | | r | 0.260** | 0.233** | 0.059 | 0.035 | 0.220** | 0.050 | 0.045 | 0.143 | 0.128 | 0.070 | 0.098 | 0.050 | -0.090 | 0.035 | 0.220** |
|  | | p | <0.001 | 0.002 | 0.444 | 0.650 | 0.004 | 0.522 | 0.564 | 0.063 | 0.096 | 0.365 | 0.203 | 0.520 | 0.244 | 0.650 | 0.004 |
| **Gaming** | | r | 0.149 | 0.113 | 0.113 | 0.117 | 0.122 | 0.165* | -0.018 | 0.123 | 0.038 | 0.047 | 0.017 | 0.005 | -0.051 | 0.109 | 0.182* |
|  | | p | 0.053 | 0.144 | 0.145 | 0.129 | 0.113 | 0.032 | 0.818 | 0.111 | 0.624 | 0.541 | 0.826 | 0.949 | 0.512 | 0.159 | 0.018 |
| **Overeating** | | r | 0.197* | 0.079 | -0.003 | 0.046 | 0.195* | 0.124 | 0.097 | 0.053 | 0.050 | 0.031 | 0.136 | -0.010 | 0.038 | 0.091 | 0.186* |
|  | | p | 0.010 | 0.304 | 0.971 | 0.549 | 0.011 | 0.107 | 0.210 | 0.496 | 0.521 | 0.687 | 0.078 | 0.899 | 0.623 | 0.238 | 0.015 |
| **Sexual**  **Activity** | | r | 0.244** | 0.245** | -0.020 | 0.076 | 0.246** | 0.196* | 0.087 | 0.105 | 0.038 | 0.084 | 0.180* | 0.136 | 0.088 | 0.158* | 0.300** |
|  |  | p | 0.002 | 0.002 | 0.796 | 0.333 | 0.002 | 0.012 | 0.269 | 0.183 | 0.626 | 0.287 | 0.021 | 0.084 | 0.264 | 0.045 | <0.001 |
| **Working** | | r | 0.249** | -0.044 | -0.042 | 0.185* | 0.182* | 0.388** | 0.520** | 0.086 | 0.078 | 0.008 | 0.248** | 0.045 | -0.136 | 0.237** | 0.315** |
|  | p | | 0.001 | 0.568 | 0.584 | 0.016 | 0.018 | <0.001 | <0.001 | 0.267 | 0.316 | 0.920 | 0.001 | 0.557 | 0.079 | 0.002 | <0.001 |

SSBA, The Brief Screener for Substance and Behavioral Addictions; DASS-21, Depression, Anxiety, Stress Scale; CSS, Chronic Stress Scale; r, Spearman’s correlation.
p-values were adjusted using the Bonferroni correction.
**p < 0.005 significant; 0.005 ≤ *p < 0.05 marginal

**Supplementary Table 5. Correlation matrix between SSBA domains and CTQ-33 domains.**

| **SSBA Domains** |  | **CTQ-33**  **Emotional Abuse** | **CTQ-33**  **Physical Abuse** | **CTQ-33**  **Sexual Abuse** | **CTQ-33**  **Emotional Neglect** | **CTQ-33**  **Physical Neglect** | **CTQ-33**  **Minimization** | **CTQ-33**  **Total** |
| --- | --- | --- | --- | --- | --- | --- | --- | --- |
| Alcohol | r | 0.186* | 0.011 | 0.124 | 0.060 | -0.154* | -0.124 | 0.102 |
|  | p | 0.015 | 0.884 | 0.107 | 0.437 | 0.046 | 0.107 | 0.188 |
| Tobacco | r | 0.133 | -0.011 | 0.177* | 0.027 | -0.030 | -0.066 | 0.078 |
|  | p | 0.086 | 0.892 | 0.022 | 0.728 | 0.701 | 0.391 | 0.312 |
| Cannabis | r | 0.226** | 0.058 | 0.137 | 0.150 | -0.011 | -0.094 | 0.159* |
|  | p | 0.003 | 0.460 | 0.077 | 0.053 | 0.883 | 0.226 | 0.040 |
| Gambling | r | -0.111 | -0.015 | -0.067 | -0.129 | -0.149 | 0.084 | -0.148 |
|  | p | 0.149 | 0.850 | 0.386 | 0.095 | 0.054 | 0.275 | 0.055 |
| Shopping | r | 0.054 | -0.022 | -0.019 | -0.056 | 0.095 | -0.017 | 0.031 |
|  | p | 0.483 | 0.777 | 0.807 | 0.473 | 0.217 | 0.827 | 0.688 |
| Gaming | r | 0.016 | -0.057 | 0.067 | -0.105 | 0.035 | 0.085 | -0.034 |
|  | p | 0.838 | 0.461 | 0.383 | 0.172 | 0.650 | 0.273 | 0.662 |
| Overeating | r | 0.073 | -0.100 | 0.036 | 0.037 | 0.005 | -0.046 | 0.073 |
|  | p | 0.346 | 0.194 | 0.645 | 0.635 | 0.951 | 0.555 | 0.348 |
| Sexual activity | r | 0.086 | 0.088 | 0.270** | 0.057 | -0.048 | -0.067 | 0.118 |
|  | p | 0.278 | 0.266 | <0.001 | 0.471 | 0.539 | 0.395 | 0.132 |
| Working | r | 0.200* | 0.110 | 0.182* | 0.064 | 0.107 | -0.051 | 0.175* |
|  | p | 0.009 | 0.153 | 0.018 | 0.409 | 0.167 | 0.513 | 0.023 |

SSBA, The Brief Screener for Substance and Behavioral Addictions; CTQ-33, Childhood Trauma Questionnaire.

r, Spearman’s correlation. p-values were adjusted using the Bonferroni correction.
**p < .005 significant; 0.005 ≤ *p < 0.05 marginal

**Supplementary Table 6. Bootstrap confidence intervals for edge weights in the Addictions Network.**

| **Node 1** | **Node 2** | **Weight** | **Mean** | **SD** | **95% CI**  **(Lower)** | **95% CI**  **(Upper)** | **CI includes**  **zero** |
| --- | --- | --- | --- | --- | --- | --- | --- |
| **SSBA-Alcohol** | SSBA-Cannabis | 0.223 | 0.237 | 0.083 | 0.069 | 0.392 | FALSE |
| **SSBA-Alcohol** | SSBA-Gambling | 0.143 | 0.232 | 0.087 | 0.061 | 0.394 | FALSE |
| **SSBA-Alcohol** | SSBA-Sexual activity | 0.177 | 0.097 | 0.075 | 0 | 0.256 | TRUE |
| **SSBA-Alcohol** | SSBA-Shopping | 0.065 | 0.152 | 0.078 | 0 | 0.310 | TRUE |
| **SSBA-Alcohol** | SSBA-Tobacco | 0.282 | 0.288 | 0.083 | 0.124 | 0.455 | FALSE |
| **SSBA-Cannabis** | SSBA-Overeating | 0.026 | -0.027 | 0.069 | -0.227 | 0.031 | TRUE |
| **SSBA-Cannabis** | SSBA-Gambling | 0.069 | -0.012 | 0.041 | -0.154 | 0.017 | TRUE |
| **SSBA-Cannabis** | SSBA-Sexual activity | 0.155 | 0.205 | 0.090 | 0.027 | 0.376 | FALSE |
| **SSBA-Cannabis** | SSBA-Tobacco | 0.229 | -0.038 | 0.088 | -0.283 | 0.067 | TRUE |
| **SSBA-Overeating** | SSBA-Sexual activity | 0.183 | 0.036 | 0.063 | 0 | 0.204 | TRUE |
| **SSBA- Overeating** | SSBA-Shopping | 0.171 | 0.194 | 0.083 | 0.023 | 0.357 | FALSE |
| **SSBA-Gambling** | SSBA-Gaming | 0.144 | 0.046 | 0.057 | 0 | 0.182 | TRUE |
| **SSBA-Gaming** | SSBA-Sexual activity | 0.161 | -0.142 | 0.139 | -0.444 | 0 | TRUE |
| **SSBA-Sexual activity** | SSBA-Tobacco | 0.032 | 0.182 | 0.094 | 0 | 0.366 | TRUE |
| **SSBA-Sexual activity** | SSBA-Working | 0.056 | 0.127 | 0.084 | 0 | 0.307 | TRUE |

SSBA, The Brief Screener for Substance and Behavioral Addictions; SD, Standard Deviation; CI: Confidence Interval.

**Supplementary Table 7. Centrality indices of the Addictions Network.**

|  | **Betweenness** | **Closeness** | **Strength** | **Expected Influence** |
| --- | --- | --- | --- | --- |
| **SSBA-Alcohol** | 11 | 0.012 | 0.889 | 0.889 |
| **SSBA-Tobacco** | 0 | 0.010 | 0.542 | 0.542 |
| **SSBA-Cannabis** | 0 | 0.011 | 0.701 | 0.701 |
| **SSBA-Gambling** | 0 | 0.008 | 0.355 | 0.355 |
| **SSBA-Shopping** | 0 | 0.007 | 0.235 | 0.235 |
| **SSBA-Gaming** | 0 | 0.009 | 0.305 | 0.305 |
| **SSBA-Eating** | 4 | 0.010 | 0.381 | 0.381 |
| **SSBA-Sexual activity** | 17 | 0.013 | 0.765 | 0.765 |
| **SSBA-Working** | 0 | 0.005 | 0.056 | 0.056 |

SSBA, The Brief Screener for Substance and Behavioral Addictions.

**Supplementary Table 8. Bridge centrality indices of the Addictions Network.**

| **Node** | **Bridge Strength** | **Bridge Betweenness** | **Bridge Closeness** |
| --- | --- | --- | --- |
| **SSBA-Alcohol** | 0.385 | 10 | 0.100 |
| **SSBA-Tobacco** | 0.032 | 0 | 0.071 |
| **SSBA-Cannabis** | 0.250 | 0 | 0.084 |
| **SSBA-Gambling** | 0.211 | 0 | 0.076 |
| **SSBA-Shopping** | 0.065 | 0 | 0.057 |
| **SSBA-Gaming** | 0 | 0 | 0.069 |
| **SSBA-Eating** | 0.210 | 4 | 0.087 |
| **SSBA-Sexual activity** | 0.604 | 17 | 0.128 |
| **SSBA-Working** | 0.056 | 0 | 0.041 |

SSBA, The Brief Screener for Substance and Behavioral Addictions.


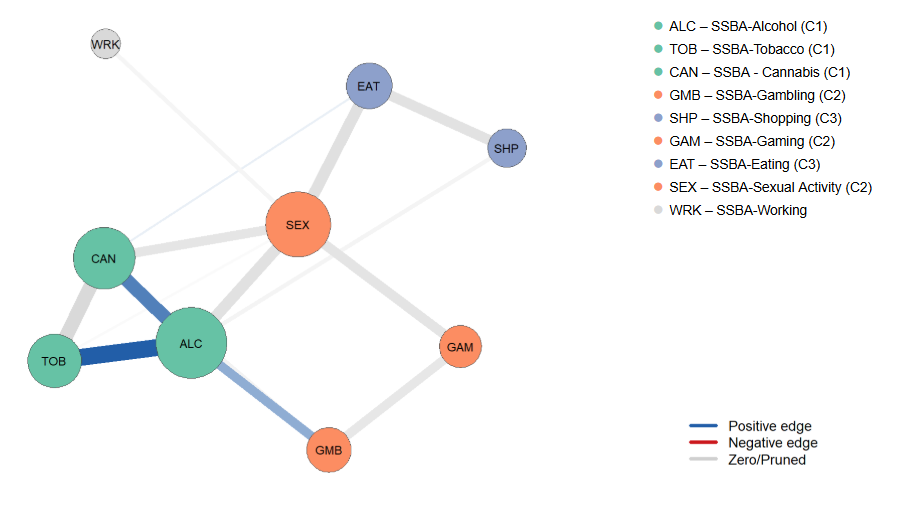
 **Supplementary Fig. 1. EGA-Derived Community Structure of the Addictions Network.** Community structure of SSBA addiction domains identified using exploratory graph analysis (EGA). The EGA-derived network was estimated using the EBICglasso algorithm (γ = 0.25). Node size reflects strength centrality, and edge thickness represents the magnitude of partial correlations. Blue edges indicate positive associations, red edges indicate negative associations, and grey edges represent pruned or near-zero associations. Node colors indicate EGA-derived community memberships.

SSBA, The Brief Screener for Substance and Behavioral Addictions.


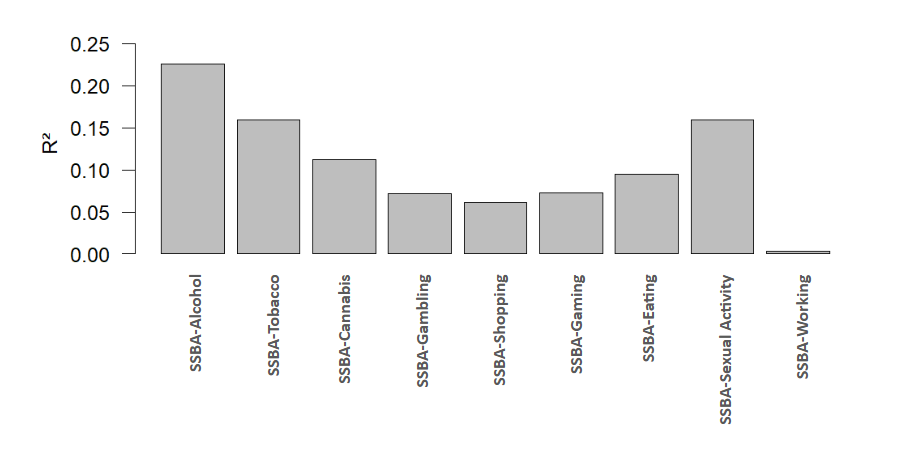


**Supplementary Fig. 2.** **The Addictions Network’s Node Predictabilities.** Node predictability for each SSBA addiction domain estimated from the EBICglasso network (γ = 0.25). Bars represent the proportion of variance in each node explained by its neighboring nodes within the network.

SSBA, The Brief Screener for Substance and Behavioral Addictions; R², Node predictability.

**Supplementary Table 9. Sensitivity analysis across different γ (EBICglasso) regularization parameters for the Addictions Network.**

| **γ** | **N**  **of edges** | **Mean absolute**  **edge weight** | **Jaccard similarity**  **vs. selected model** | **CS-coefficient**  **(Strength)** | **CS-coefficient**  **(Expected Influence)** |
| --- | --- | --- | --- | --- | --- |
| 0 | 19 | 0.0728 | 0.789 | NA | NA |
| 0.25 | 15 | 0.0587 | 1.000 | NA | NA |
| 0.50 | 15 | 0.0587 | 1.000 | NA | NA |
| 0.75 | 15 | 0.0587 | 1.000 | NA | NA |
| 1.00 | 15 | 0.0587 | 1.000 | NA | NA |

γ, Gamma; N, Number; CS, Correlation Stability.

**Supplementary Table 10. Bootstrap confidence intervals for edge weights in the Addictions and Psychometric Features Network.**

| **Node1** | **Node2** | **Weight** | **Mean** | **SD** | **95% CI**  **(Lower)** | **95% CI**  **(Upper)** | **CI includes**  **zero** |
| --- | --- | --- | --- | --- | --- | --- | --- |
| **Anhedonia (SHAPS)** | **Depression (DASS-21)** | 0.157 | 0.194 | 0.060 | 0.073 | 0.302 | FALSE |
| **Anxiety (DASS-21)** | **Depression (DASS-21)** | 0.323 | 0.014 | 0.031 | 0 | 0.101 | TRUE |
| **Anxiety (DASS-21)** | **Impulsivity (BIS-11)** | 0.115 | 0.057 | 0.052 | 0 | 0.169 | TRUE |
| **Anxiety (DASS-21)** | **SSBA-Alcohol** | 0.077 | 0.153 | 0.085 | 0 | 0.317 | TRUE |
| **Anxiety (DASS-21)** | **SSBA-Shopping** | 0.024 | -0.081 | 0.070 | -0.222 | 0 | TRUE |
| **Anxiety (DASS-21)** | **SSBA-Tobacco** | 0.070 | 0.164 | 0.080 | 0 | 0.318 | TRUE |
| **Anxiety (DASS-21)** | **Stress (DASS-21)** | 0.297 | 0.307 | 0.065 | 0.181 | 0.435 | FALSE |
| **Childhood Trauma** | **Chronic Stress** | 0.056 | 0.008 | 0.023 | 0 | 0.086 | TRUE |
| **Childhood Trauma** | **Impulsivity (BIS-11)** | 0.069 | 0.034 | 0.043 | 0 | 0.142 | TRUE |
| **Chronic Stress** | **Depression (DASS-21)** | 0.114 | 0.113 | 0.060 | 0 | 0.229 | TRUE |
| **Chronic Stress** | **SSBA-Tobacco** | 0.012 | 0.031 | 0.046 | 0 | 0.150 | TRUE |
| **Chronic Stress** | **SSBA-Working** | 0.042 | 0.076 | 0.055 | 0 | 0.192 | TRUE |
| **Chronic Stress** | **Stress (DASS-21)** | 0.188 | 0.330 | 0.058 | 0.209 | 0.438 | FALSE |
| **Depression (DASS-21)** | **Impulsivity (BIS-11)** | 0.013 | 0.018 | 0.033 | 0 | 0.111 | TRUE |
| **Depression (DASS-21)** | **SSBA-Alcohol** | 0.051 | -0.012 | 0.054 | -0.167 | 0.059 | TRUE |
| **Depression (DASS-21)** | **SSBA-Tobacco** | 0.032 | 0.128 | 0.076 | 0 | 0.279 | TRUE |
| **Depression (DASS-21)** | **Stress (DASS-21)** | 0.253 | 0.261 | 0.064 | 0.134 | 0.385 | FALSE |
| **Impulsivity (BIS-11)** | **SSBA-Alcohol** | 0.120 | 0.007 | 0.026 | -0.013 | 0.086 | TRUE |
| **Impulsivity (BIS-11)** | **SSBA-Cannabis** | 0.082 | 0.035 | 0.044 | 0 | 0.144 | TRUE |
| **Impulsivity (BIS-11)** | **SSBA-Eating** | 0.040 | 0.039 | 0.046 | 0 | 0.149 | TRUE |
| **Impulsivity (BIS-11)** | **SSBA-Gaming** | 0.085 | -0.015 | 0.034 | -0.122 | 0 | TRUE |
| **Impulsivity (BIS-11)** | **SSBA- Sexual activity** | 0.016 | 0.121 | 0.067 | 0 | 0.251 | TRUE |
| **Impulsivity (BIS-11)** | **SSBA-Shopping** | 0.111 | 0.108 | 0.067 | 0 | 0.238 | TRUE |
| **Impulsivity (BIS-11)** | **SSBA-Tobacco** | 0.109 | 0.084 | 0.080 | 0 | 0.260 | TRUE |
| **SSBA-Alcohol** | **SSBA-Cannabis** | 0.190 | 0.204 | 0.078 | 0.049 | 0.354 | FALSE |
| **SSBA-Alcohol** | **SSBA-Gambling** | 0.105 | 0.199 | 0.078 | 0.044 | 0.346 | FALSE |
| **SSBA-Alcohol** | **SSBA- Sexual activity** | 0.143 | 0.035 | 0.048 | 0 | 0.159 | TRUE |
| **SSBA-Alcohol** | **SSBA-Tobacco** | 0.209 | 0.215 | 0.075 | 0.064 | 0.358 | FALSE |
| **SSBA-Alcohol** | **Stress (DASS-21)** | 0.017 | 0.015 | 0.036 | 0 | 0.120 | TRUE |
| **SSBA-Cannabis** | **SSBA-Gambling** | 0.036 | -0.013 | 0.032 | -0.112 | 0 | TRUE |
| **SSBA-Cannabis** | **SSBA- Sexual activity** | 0.132 | 0.026 | 0.047 | 0 | 0.152 | TRUE |
| **SSBA-Cannabis** | **SSBA-Tobacco** | 0.186 | -0.005 | 0.023 | -0.073 | 0 | TRUE |
| **SSBA-Eating** | **SSBA- Sexual activity** | 0.133 | 0.022 | 0.038 | 0 | 0.126 | TRUE |
| **SSBA-Eating** | **SSBA-Shopping** | 0.105 | 0.101 | 0.064 | 0 | 0.226 | TRUE |
| **SSBA-Gambling** | **SSBA-Gaming** | 0.087 | 0.027 | 0.041 | 0 | 0.133 | TRUE |
| **SSBA-Gaming** | **SSBA- Sexual activity** | 0.103 | 0.005 | 0.017 | 0 | 0.059 | TRUE |
| **SSBA- Sexual activity** | **SSBA-Tobacco** | 0.006 | 0.011 | 0.035 | -0.012 | 0.111 | TRUE |
| **SSBA-Sexual activity** | **Stress (DASS-21)** | 0.056 | 0.087 | 0.063 | 0 | 0.217 | TRUE |
| **SSBA-Shopping** | **Stress (DASS-21)** | 0.004 | 0.009 | 0.023 | 0 | 0.075 | TRUE |
| **SSBA-Working** | **Stress (DASS-21)** | 0.016 | -0.001 | 0.017 | -0.029 | 0.019 | TRUE |

BIS-11, Barratt Impulsiveness Scale-11; CTQ-33, Childhood Trauma Questionnaire; CSS, Chronic Stress Scale; DASS-21, Depression Anxiety Stress Scales-21; SHAPS, Snaith-Hamilton Pleasure Scale; SSBA, The Brief Screener for Substance and Behavioral Addictions; SD, Standard Deviation; CI, Confidence Interval.

**Supplementary Table 11. Centrality indices of the Addictions and Psychometric Features Network.**

|  | **Betweenness** | **Closeness** | **Strength** | **Expected Influence** |
| --- | --- | --- | --- | --- |
| **SSBA-Alcohol** | 24 | 0.004 | 0.913 | 0.913 |
| **SSBA-Tobacco** | 0 | 0.004 | 0.625 | 0.625 |
| **SSBA-Cannabis** | 0 | 0.003 | 0.627 | 0.627 |
| **SSBA-Gambling** | 0 | 0.003 | 0.228 | 0.228 |
| **SSBA-Shopping** | 3 | 0.003 | 0.244 | 0.244 |
| **SSBA-Gaming** | 0 | 0.003 | 0.275 | 0.275 |
| **SSBA-Eating** | 1 | 0.003 | 0.279 | 0.279 |
| **SSBA-** **Sexual activity** | 12 | 0.004 | 0.589 | 0.589 |
| **SSBA-Working** | 0 | 0.002 | 0.057 | 0.057 |
| **Chronic Stress** | 15 | 0.003 | 0.412 | 0.412 |
| **Childhood Trauma** | 0 | 0.003 | 0.125 | 0.125 |
| **Impulsivity (BIS-11)** | 30 | 0.004 | 0.761 | 0.761 |
| **Anhedonia (SHAPS)** | 0 | 0.003 | 0.157 | 0.157 |
| **Depression (DASS-21)** | 14 | 0.004 | 0.942 | 0.942 |
| **Anxiety (DASS-21)** | 37 | 0.004 | 0.904 | 0.904 |
| **Stress (DASS-21)** | 24 | 0.004 | 0.829 | 0.829 |

BIS-11, Barratt Impulsiveness Scale-11; CTQ-33, Childhood Trauma Questionnaire; CSS, Chronic Stress Scale; DASS-21, Depression Anxiety Stress Scales-21; SHAPS, Snaith-Hamilton Pleasure Scale; SSBA, The Brief Screener for Substance and Behavioral Addictions; SD, Standard Deviation.


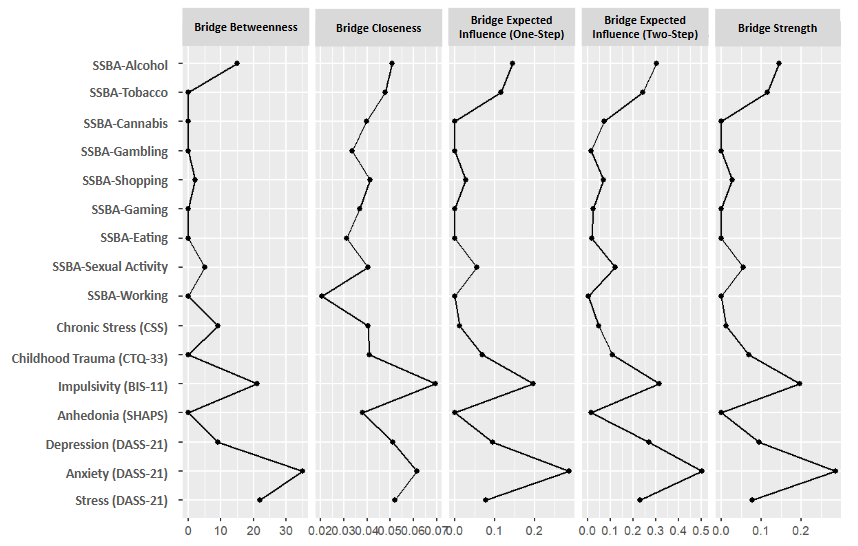


**Supplementary Fig. 3. Bridge Centrality Indices of the Addictions and Psychometric Features Network.** Bridge centrality indices for SSBA addiction domains and clinical variables are estimated from the EBICglasso network. Panels display bridge betweenness, bridge closeness, bridge expected influence (one-step and two-step), and bridge strength, indicating the relative contribution of each node to connections between network communities.

BIS-11, Barratt Impulsiveness Scale-11; CTQ-33, Childhood Trauma Questionnaire; CSS, Chronic Stress Scale; DASS-21, Depression Anxiety Stress Scales-21; SHAPS, Snaith-Hamilton Pleasure Scale; SSBA, The Brief Screener for Substance and Behavioral Addictions; SD, Standard Deviation.

**Supplementary Table 12. Bridge centrality indices of the Addictions and Psychometric Features Network.**

| **Node** | **Bridge Strength** | **Bridge Betweenness** | **Bridge Closeness** |
| --- | --- | --- | --- |
| **SSBA-Alcohol** | 0.144 | 15 | 0.051 |
| **SSBA-Tobacco** | 0.114 | 0 | 0.048 |
| **SSBA-Cannabis** | 0 | 0 | 0.040 |
| **SSBA-Gambling** | 0 | 0 | 0.033 |
| **SSBA-Shopping** | 0.028 | 2 | 0.041 |
| **SSBA-Gaming** | 0 | 0 | 0.037 |
| **SSBA-Eating** | 0 | 0 | 0.031 |
| **SSBA-Sexual activity** | 0.056 | 5 | 0.041 |
| **SSBA-Working** | 0 | 0 | 0.020 |
| **Chronic Stress** | 0.012 | 9 | 0.040 |
| **Childhood Trauma** | 0.069 | 0 | 0.041 |
| **Impulsivity (BIS-11)** | 0.196 | 21 | 0.070 |
| **Anhedonia (SHAPS)** | 0 | 0 | 0.038 |
| **Depression (DASS-21)** | 0.095 | 9 | 0.051 |
| **Anxiety (DASS-21)** | 0.285 | 35 | 0.061 |
| **Stress (DASS-21)** | 0.077 | 22 | 0.052 |

BIS-11, Barratt Impulsiveness Scale-11; CTQ-33, Childhood Trauma Questionnaire; CSS, Chronic Stress Scale; DASS-21, Depression Anxiety Stress Scales-21; SHAPS, Snaith-Hamilton Pleasure Scale; SSBA, The Brief Screener for Substance and Behavioral Addictions; SD, Standard Deviation.


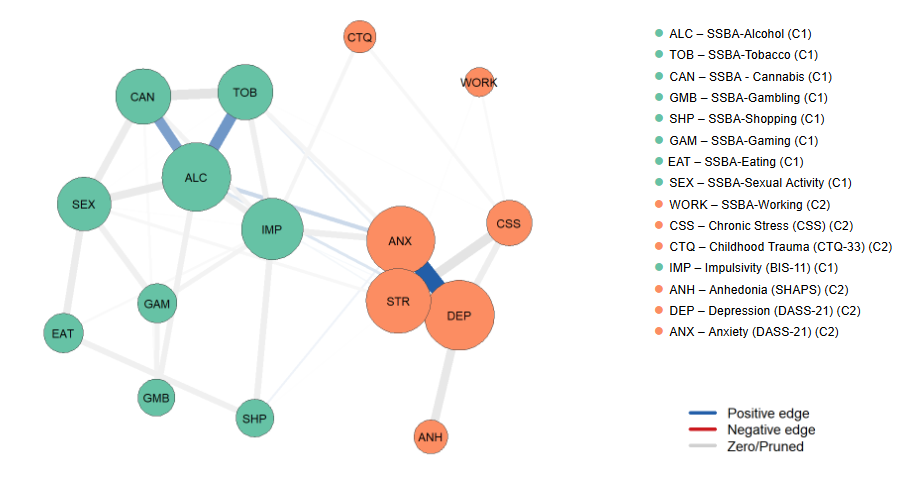


**Supplementary Fig. 4. EGA-Derived Community Structure of the Addictions and Psychometric Features Network.** Community structure of SSBA addiction domains and clinical variables identified using exploratory graph analysis (EGA). The EGA-derived network was estimated using the EBICglasso algorithm (γ = 0.25). Node size reflects strength centrality, and edge thickness represents the magnitude of partial correlations. Blue edges indicate positive associations, red edges indicate negative associations, and grey edges represent pruned or near-zero associations. Node colors indicate EGA-derived community memberships.

BIS-11, Barratt Impulsiveness Scale-11; CTQ-33, Childhood Trauma Questionnaire; CSS, Chronic Stress Scale; DASS-21, Depression Anxiety Stress Scales-21; SHAPS, Snaith-Hamilton Pleasure Scale; SSBA, The Brief Screener for Substance and Behavioral Addictions.


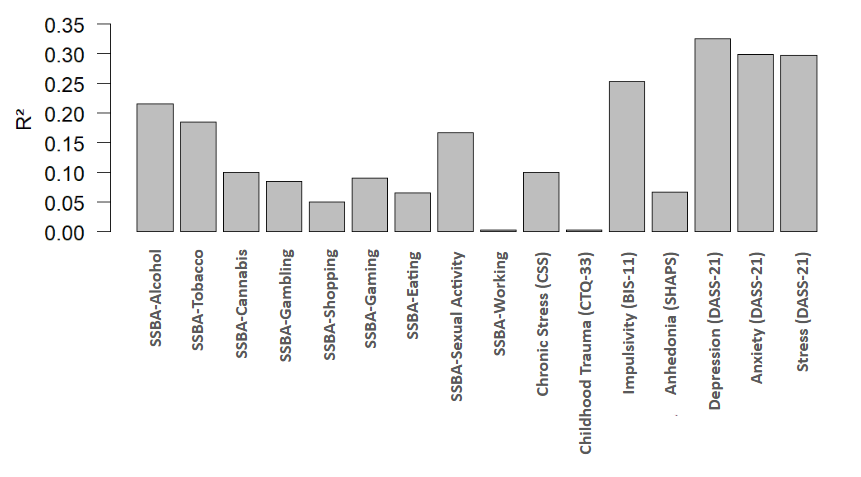


**Supplementary Fig. 5. The Addictions and Psychometric Features Network’s Node Predictabilities.** Node predictability for SSBA addiction domains and clinical variables estimated from the EBICglasso network (γ = 0.25). Bars represent the proportion of variance in each node explained by its neighboring nodes within the network.

BIS-11, Barratt Impulsiveness Scale-11; CTQ-33, Childhood Trauma Questionnaire; CSS, Chronic Stress Scale; DASS-21, Depression Anxiety Stress Scales-21; SHAPS, Snaith-Hamilton Pleasure Scale; SSBA, The Brief Screener for Substance and Behavioral Addictions; R², Node predictability.

**Supplementary Table 13. Sensitivity analysis across different γ (EBICglasso) regularization parameters for the Addictions and Psychometric Features Network.**

| **γ** | **N**  **of edges** | **Mean absolute**  **edge weight** | **Jaccard similarity**  **vs. selected model** | **CS-coefficient**  **(Strength)** | **CS-coefficient**  **(Expected Influence)** |
| --- | --- | --- | --- | --- | --- |
| 0 | 59 | 0.0480 | 0.678 | NA | NA |
| 0.25 | 40 | 0.0332 | 1.000 | NA | NA |
| 0.50 | 40 | 0.0332 | 1.000 | NA | NA |
| 0.75 | 8 | 0.0068 | 0.200 | NA | NA |
| 1.00 | 8 | 0.0068 | 0.200 | NA | NA |

γ, Gamma; N, Number; CS, Correlation Stability.


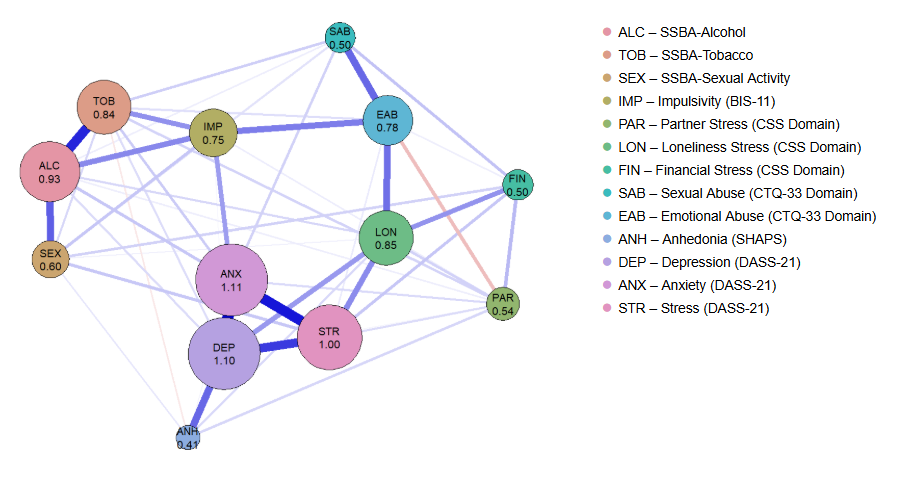


**Supplementary Fig. 6. The Focused Addictions and Psychometric Features Network.** Network structure of SSBA addiction domains, impulsivity, anhedonia, childhood trauma subdomains, and chronic stress domains estimated using the EBICglasso algorithm (γ = 0.25). Node size reflects strength centrality. Edge thickness reflects the magnitude of partial correlations; blue edges indicate positive associations, red edges indicate negative associations, and grey edges represent pruned or near-zero associations.

BIS-11, Barratt Impulsiveness Scale-11; CTQ-33, Childhood Trauma Questionnaire; CSS, Chronic Stress Scale; DASS-21, Depression Anxiety Stress Scales-21; SHAPS, Snaith-Hamilton Pleasure Scale; SSBA, The Brief Screener for Substance and Behavioral Addictions.

**Supplementary Table 14. Bootstrap confidence intervals for edge weights in the Focused Addictions and Psychometric Features Network.**

| **Node1** | **Node2** | **Weight** | **Mean** | **SD** | **95% CI**  **(Lower)** | **95% CI**  **(Upper)** | **CI Includes**  **zero** |
| --- | --- | --- | --- | --- | --- | --- | --- |
| **Anhedonia (SHAPS)** | Anxiety (DASS-21) | 0.016 | 0.005 | 0.017 | 0 | 0.063 | TRUE |
| **Anhedonia (SHAPS)** | Depression (DASS-21) | 0.212 | 0.062 | 0.051 | 0 | 0.178 | TRUE |
| **Anhedonia (SHAPS)** | Loneliness Stress (CSS) | 0.054 | 0.008 | 0.023 | 0 | 0.080 | TRUE |
| **Anhedonia (SHAPS)** | Partner Stress (CSS) | 0.057 | 0.043 | 0.049 | 0 | 0.160 | TRUE |
| **Anhedonia (SHAPS)** | SSBA-Sexual activity | 0.035 | 0.043 | 0.052 | 0 | 0.168 | TRUE |
| **Anhedonia (SHAPS)** | SSBA-Tobacco | -0.033 | 0.074 | 0.068 | 0 | 0.224 | TRUE |
| **Anxiety (DASS-21)** | Depression (DASS-21) | 0.356 | 0.013 | 0.030 | 0 | 0.102 | TRUE |
| **Anxiety (DASS-21)** | Impulsivity (BIS-11) | 0.137 | 0.049 | 0.061 | 0 | 0.195 | TRUE |
| **Anxiety (DASS-21)** | Partner Stress (CSS) | 0.053 | 0.023 | 0.035 | 0 | 0.117 | TRUE |
| **Anxiety (DASS-21)** | Sexual Abuse (CTQ-33) | 0.065 | 0.028 | 0.040 | 0 | 0.139 | TRUE |
| **Anxiety (DASS-21)** | SSBA-Alcohol | 0.079 | 0.046 | 0.050 | 0 | 0.165 | TRUE |
| **Anxiety (DASS-21)** | SSBA-Tobacco | 0.073 | 0.036 | 0.047 | 0 | 0.153 | TRUE |
| **Anxiety (DASS-21)** | Stress (DASS-21) | 0.327 | 0.309 | 0.068 | 0.177 | 0.441 | FALSE |
| **Depression (DASS-21)** | Impulsivity (BIS-11) | 0.010 | 0.011 | 0.029 | 0 | 0.099 | TRUE |
| **Depression (DASS-21)** | Loneliness Stress (CSS) | 0.142 | 0.172 | 0.063 | 0.045 | 0.296 | FALSE |
| **Depression (DASS-21)** | Partner Stress (CSS) | 0.013 | 0.010 | 0.023 | 0 | 0.077 | TRUE |
| **Depression (DASS-21)** | SSBA-Alcohol | 0.050 | 0.019 | 0.032 | 0 | 0.106 | TRUE |
| **Depression (DASS-21)** | SSBA-Tobacco | 0.049 | 0.125 | 0.068 | 0 | 0.263 | TRUE |
| **Depression (DASS-21)** | Stress (DASS-21) | 0.274 | 0.264 | 0.064 | 0.140 | 0.390 | FALSE |
| **Emotional Abuse (CTQ-33)** | Financial Stress (CSS) | 0.024 | 0.114 | 0.061 | 0 | 0.231 | TRUE |
| **Emotional Abuse (CTQ-33)** | Impulsivity (BIS-11) | 0.181 | -0.024 | 0.044 | -0.146 | 0 | TRUE |
| **Emotional Abuse (CTQ-33)** | Loneliness Stress (CSS) | 0.200 | -0.002 | 0.019 | -0.058 | 0.022 | TRUE |
| **Emotional Abuse (CTQ-33)** | Partner Stress (CSS) | -0.092 | 0.041 | 0.041 | 0 | 0.137 | TRUE |
| **Emotional Abuse (CTQ-33)** | Sexual Abuse (CTQ-33) | 0.212 | 0.008 | 0.022 | 0 | 0.073 | TRUE |
| **Emotional Abuse (CTQ-33)** | SSBA-Tobacco | 0.046 | 0.009 | 0.026 | 0 | 0.091 | TRUE |
| **Emotional Abuse (CTQ-33)** | Stress (DASS-21) | 0.029 | 0.150 | 0.057 | 0.037 | 0.257 | FALSE |
| **Financial Stress (CSS)** | Loneliness Stress (CSS) | 0.151 | 0.134 | 0.071 | 0 | 0.279 | TRUE |
| **Financial Stress (CSS)** | Partner Stress (CSS) | 0.098 | 0.005 | 0.021 | 0 | 0.071 | TRUE |
| **Financial Stress (CSS)** | Sexual Abuse (CTQ-33) | 0.085 | -0.001 | 0.016 | -0.047 | 0.016 | TRUE |
| **Financial Stress (CSS)** | SSBA-Sexual activity | 0.065 | 0.004 | 0.016 | 0 | 0.058 | TRUE |
| **Financial Stress (CSS)** | Stress (DASS-21) | 0.080 | 0.060 | 0.063 | 0 | 0.200 | TRUE |
| **Impulsivity (BIS-11)** | Partner Stress (CSS) | 0.026 | 0.164 | 0.063 | 0.038 | 0.288 | FALSE |
| **Impulsivity (BIS-11)** | SSBA-Alcohol | 0.166 | 0.059 | 0.062 | 0 | 0.202 | TRUE |
| **Impulsivity (BIS-11)** | SSBA-Sexual activity | 0.079 | 0.039 | 0.049 | 0 | 0.161 | TRUE |
| **Impulsivity (BIS-11)** | SSBA-Tobacco | 0.154 | 0.017 | 0.031 | 0 | 0.110 | TRUE |
| **Loneliness Stress (CSS)** | Partner Stress (CSS) | 0.063 | 0.012 | 0.029 | 0 | 0.102 | TRUE |
| **Loneliness Stress (CSS)** | SSBA-Alcohol | 0.057 | 0.146 | 0.073 | 0 | 0.285 | TRUE |
| **Loneliness Stress (CSS)** | SSBA-Sexual activity | 0.022 | 0.002 | 0.029 | -0.068 | 0.081 | TRUE |
| **Loneliness Stress (CSS)** | Stress (DASS-21) | 0.162 | 0.067 | 0.054 | 0 | 0.181 | TRUE |
| **Partner Stress (CSS)** | SSBA-Alcohol | 0.025 | 0.153 | 0.072 | 0 | 0.289 | TRUE |
| **Partner Stress (CSS)** | SSBA-Tobacco | 0.061 | 0.052 | 0.064 | 0 | 0.216 | TRUE |
| **Partner Stress (CSS)** | Stress (DASS-21) | 0.050 | 0.025 | 0.037 | 0 | 0.124 | TRUE |
| **Sexual Abuse (CTQ-33)** | SSBA-Alcohol | 0.025 | 0.030 | 0.045 | 0 | 0.152 | TRUE |
| **Sexual Abuse (CTQ-33)** | SSBA-Sexual activity | 0.041 | 0.061 | 0.054 | 0 | 0.176 | TRUE |
| **Sexual Abuse (CTQ-33)** | SSBA-Tobacco | 0.069 | 0.048 | 0.051 | 0 | 0.168 | TRUE |
| **SSBA-Alcohol** | SSBA-Sexual activity | 0.224 | 0.195 | 0.071 | 0.057 | 0.334 | FALSE |
| **SSBA-Alcohol** | SSBA-Tobacco | 0.304 | 0.282 | 0.077 | 0.125 | 0.423 | FALSE |
| **SSBA-Alcohol** | Stress (DASS-21) | 0.003 | 0.018 | 0.033 | 0 | 0.117 | TRUE |
| **SSBA-Sexual activity** | SSBA-Tobacco | 0.056 | 0.033 | 0.046 | 0 | 0.152 | TRUE |
| **SSBA-Sexual activity** | Stress (DASS-21) | 0.078 | -0.053 | 0.058 | -0.187 | 0 | TRUE |

Note: r: Spearman’s correlation, *p<0.05, **p<0.001

BIS-11, Barratt Impulsiveness Scale-11; CTQ-33, Childhood Trauma Questionnaire; CSS, Chronic Stress Scale; DASS-21, Depression Anxiety Stress Scales-21; SHAPS, Snaith-Hamilton Pleasure Scale; SSBA, The Brief Screener for Substance and Behavioral Addictions.


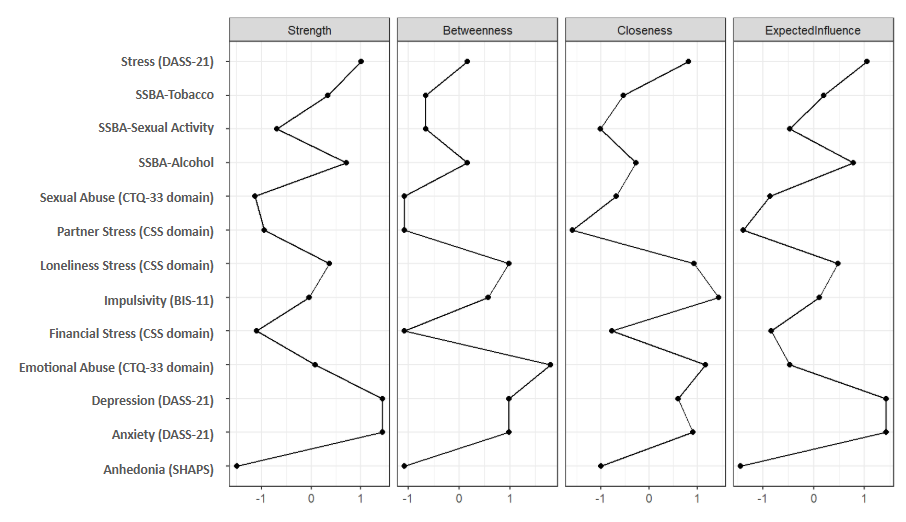


**Supplementary Fig. 7. Centrality Indices of the Focused Addictions and Psychometric Features Network.** Centrality indices of SSBA addiction domains and clinical variables are estimated from the partial correlation network. Panels display strength, betweenness, closeness, and expected influence.

BIS-11, Barratt Impulsiveness Scale-11; CTQ-33, Childhood Trauma Questionnaire; CSS, Chronic Stress Scale; DASS-21, Depression Anxiety Stress Scales-21; SHAPS, Snaith-Hamilton Pleasure Scale; SSBA, The Brief Screener for Substance and Behavioral Addictions.

**Supplementary Table 15. Centrality indices for the Focused Addictions and Psychometric Features Network.**

|  | **Betweenness** | **Closeness** | **Strength** | **Expected Influence** |
| --- | --- | --- | --- | --- |
| **SSBA-Alcohol** | 6 | 0.006 | 0.934 | 0.934 |
| **SSBA-Tobacco** | 2 | 0.006 | 0.844 | 0.778 |
| **SSBA-Sexual activity** | 2 | 0.005 | 0.600 | 0.600 |
| **Impulsivity (BIS-11)** | 8 | 0.008 | 0.753 | 0.753 |
| **Partner Stress (CSS domain)** | 0 | 0.005 | 0.538 | 0.354 |
| **Loneliness Stress (CSS domain)** | 10 | 0.007 | 0.851 | 0.851 |
| **Financial Stress (CSS domain)** | 0 | 0.006 | 0.502 | 0.502 |
| **Sexual Abuse (CTQ-33 domain)** | 0 | 0.006 | 0.496 | 0.496 |
| **Emotional Abuse (CTQ-33 domain)** | 14 | 0.008 | 0.783 | 0.599 |
| **Anhedonia (SHAPS)** | 0 | 0.005 | 0.407 | 0.341 |
| **Depression (DASS-21)** | 10 | 0.007 | 1.105 | 1.105 |
| **Anxiety (DASS-21)** | 10 | 0.007 | 1.106 | 1.106 |
| **Stress (DASS-21)** | 6 | 0.007 | 1.004 | 1.004 |

BIS-11, Barratt Impulsiveness Scale-11; CTQ-33, Childhood Trauma Questionnaire; CSS, Chronic Stress Scale; DASS-21, Depression Anxiety Stress Scales-21; SHAPS, Snaith-Hamilton Pleasure Scale; SSBA, The Brief Screener for Substance and Behavioral Addictions.


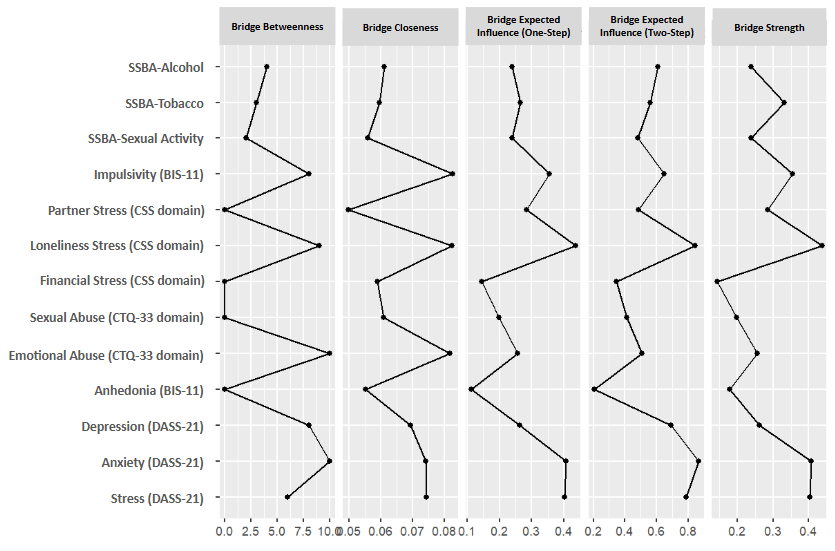


**Supplementary Fig. 8. Bridge Centrality Indices of the Focused Addictions and Psychometric Features Network.** Bridge centrality indices of SSBA addiction domains and focused clinical variables are estimated from the partial correlation network. Panels display bridge betweenness, bridge closeness, bridge expected influence (one-step and two-step), and bridge strength, reflecting the relative contribution of each node to connections between network communities.

BIS-11, Barratt Impulsiveness Scale-11; CTQ-33, Childhood Trauma Questionnaire; CSS, Chronic Stress Scale; DASS-21, Depression Anxiety Stress Scales-21; SHAPS, Snaith-Hamilton Pleasure Scale; SSBA, The Brief Screener for Substance and Behavioral Addictions.

**Supplementary Table 16. Bridge centrality indices of the Focused Addictions and Psychometric Features Network.**

| **Node** | **Bridge Strength** | **Bridge Betweenness** | **Bridge Closeness** |
| --- | --- | --- | --- |
| **SSBA-Alcohol** | 0.239 | 4 | 0.061 |
| **SSBA-Tobacco** | 0.331 | 3 | 0.060 |
| **SSBA-Sexual activity** | 0.240 | 2 | 0.056 |
| **Impulsivity (BIS-11)** | 0.354 | 8 | 0.083 |
| **Partner Stress (CSS domain)** | 0.285 | 0 | 0.050 |
| **Loneliness Stress (CSS domain)** | 0.437 | 9 | 0.083 |
| **Financial Stress (CSS domain)** | 0.145 | 0 | 0.059 |
| **Sexual Abuse (CTQ-33 domain)** | 0.199 | 0 | 0.061 |
| **Emotional Abuse (CTQ-33 domain)** | 0.256 | 10 | 0.082 |
| **Anhedonia (SHAPS)** | 0.179 | 0 | 0.055 |
| **Depression (DASS-21)** | 0.263 | 8 | 0.069 |
| **Anxiety (DASS-21)** | 0.407 | 10 | 0.074 |
| **Stress (DASS-21)** | 0.402 | 6 | 0.074 |

BIS-11, Barratt Impulsiveness Scale-11; CTQ-33, Childhood Trauma Questionnaire; CSS, Chronic Stress Scale; DASS-21, Depression Anxiety Stress Scales-21; SHAPS, Snaith-Hamilton Pleasure Scale; SSBA, The Brief Screener for Substance and Behavioral Addictions.


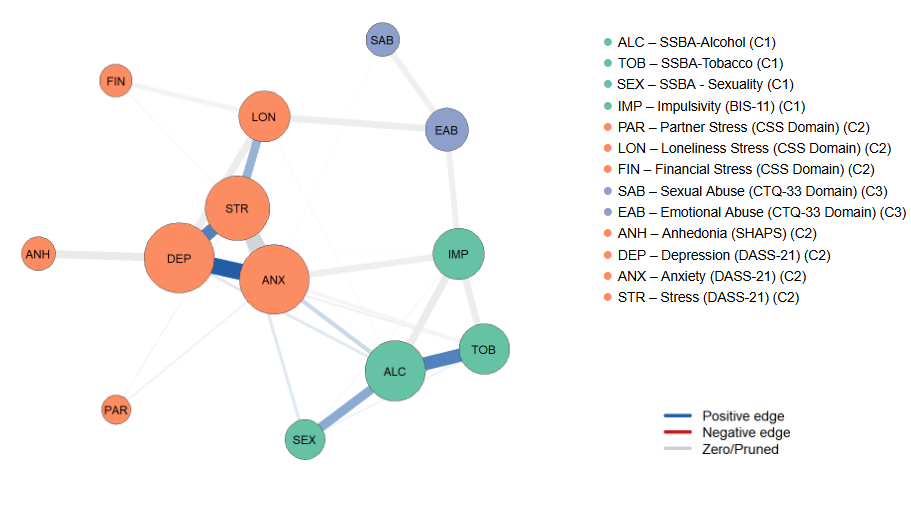


**Supplementary Fig. 9. EGA-Derived Community Structure of the Focused Addictions and Psychometric Features Network.** Community structure of the focused network identified using exploratory graph analysis (EGA). The EGA-derived network was estimated using the EBICglasso algorithm (γ = 0.25). Node size reflects strength centrality, and edge thickness represents the magnitude of partial correlations. Blue edges indicate positive associations, red edges indicate negative associations, and grey edges represent pruned or near-zero associations. Node colors indicate EGA-derived community memberships.

BIS-11, Barratt Impulsiveness Scale-11; CTQ-33, Childhood Trauma Questionnaire; CSS, Chronic Stress Scale; DASS-21, Depression Anxiety Stress Scales-21; SHAPS, Snaith-Hamilton Pleasure Scale; SSBA, The Brief Screener for Substance and Behavioral Addictions.


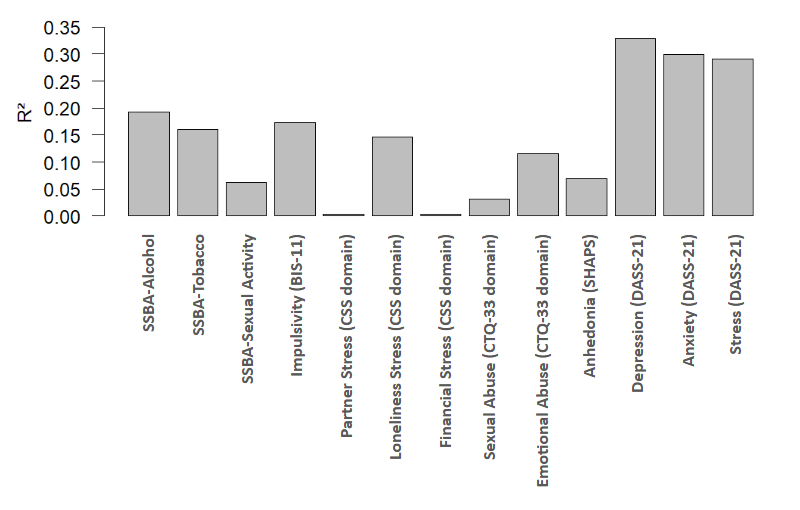


**Supplementary Fig. 10. The Focused Addictions and Psychometric Features Network’s Node Predictabilities.** Node predictability for SSBA addiction domains and focused clinical variables estimated from the EBICglasso network (γ = 0.25). Bars represent the proportion of variance in each node explained by its neighboring nodes within the network.

BIS-11, Barratt Impulsiveness Scale-11; CTQ-33, Childhood Trauma Questionnaire; CSS, Chronic Stress Scale; DASS-21, Depression Anxiety Stress Scales-21; SHAPS, Snaith-Hamilton Pleasure Scale; SSBA, The Brief Screener for Substance and Behavioral Addictions; R², Node predictability.

**Supplementary Table 17. Sensitivity analysis across different γ (EBICglasso) regularization parameters for the Focused Addictions and Psychometric Features Network.**

| **γ** | **N**  **of edges** | **Mean absolute**  **edge weight** | **Jaccard similarity**  **vs. selected model** | **CS-coefficient**  **(Strength)** | **CS-coefficient**  **(Expected Influence)** |
| --- | --- | --- | --- | --- | --- |
| 0 | 50 | 0.064 | 1.000 | NA | NA |
| 0.25 | 50 | 0.064 | 1.000 | NA | NA |
| 0.50 | 19 | 0.025 | 0.380 | NA | NA |
| 0.75 | 19 | 0.025 | 0.380 | NA | NA |
| 1.00 | 4 | 0.007 | 0.080 | NA | NA |

γ; Gamma, N; Number; CS, Correlation Stability.
